# Enhanced multimodal transformer framework for sarcasm detection with particle swarm optimization based feature fusion

**DOI:** 10.3389/frai.2026.1947291

**Published:** 2026-10-22

**Authors:** Vansh Kumar Payala, Avi Aaryan Jeet, S. Abinaya

**Affiliations:** School of Computer Science and Engineering, Vellore Institute of Technology, Chennai, India

**Keywords:** feature fusion, HuBERT, human–computer interaction, multimodal learning, particle swarm optimization, RoBERTa, sarcasm detection, transformer models

## Abstract

Intelligent Human-Computer Interaction (HCI) has a pivotal place in sarcasm detection, given that sarcastic utterances may have an intended meaning that is not in the literal meaning of the spoken words. Sarcasm is also difficult to spot correctly since it varies not only in text but also in voice and other visual indicators like facial expression and body language. Unimodal sarcasm detection methods are generally limited by contextual ambiguity, the insensitivity of contextual paralinguistic cues, and decreased performance in situations where one modality is noisy or incomplete. These problems may cause unpredictable results and inadequate functionality in the real conversational context. To overcome these shortcomings, for sarcasm detection, we suggest an approach with different modalities which uses multi-modal cues like audio, video as well as text to provide complementary information from various communication channels. In the case of textual representation, the RoBERTa architecture was used to elicit deep contextual and semantic features of dialog transcripts. The HuBERT architecture was used to model acoustic features and is effective at representing speech representations in terms of prosodic and tonal variation based on sarcastic speech. Simultaneously, the ViViT architecture was used to process visual data and identify both spatial and temporal patterns of facial expressions and gestures in videos. Modality-specific feature representations are then combined with a Particle Swarm Optimization (PSO) based fusion strategy which decides best-found modality weights by minimizing classification loss. The fused representation is then optimized and then through the two-layer fully connected classifier to classify sarcasm. The suggested framework was evaluated with the publicly available MUStARD dataset. The model displayed an accuracy of 94% and the recall, precision and weighted F1-score were all equal to 0.94. Comparative evaluation also compared the proposed PSO-Multimodal Fusion Model with the unimodal models and also with the traditional multimodal fusion models such as attention-based model, Graph-based model, Generative Approach, LLM/Modeling-based model, and contrastive learning approach, and showed that the proposed multimodal fusion framework yields the highest weighted F1-score among the evaluated approaches. Experimental outcomes prove that the multimodal fusion methodology based on optimization enhances the performance of sarcasm detection in contrast to the unimodal and traditional fusion techniques, which indicate its efficiency in practical use in conversational AI.

## 1 Introduction

Detection of sarcasm is also an important task in enhancing intelligent human-computer interaction (HCI) systems since sarcasm frequently carries a connotation that is antithetical to their literal meaning. Sarcasm in the daily conversation is often conveyed using minor linguistic signals, intonation, facial expressions and contextual knowledge. Conversational AI, virtual assistants, sentiment analysis, and social media monitoring platforms can better understand the user’s intent when it is conveyed in a sarcastic manner, allowing them to respond with more contextually relevant and human-like content. This has brought great focus on sarcasm detection in various research fields, including NLP, affective computing, and multimodal ML.

There are a lot of different variations of figurative speech, such as “sarcasm,” which is a figure of speech that requires the listener to pick up the tone to determine the meaning, and therefore is difficult for any intelligent system to automatically interpret. Applications like sentiment, social media systems monitoring, opinion mining, conversational agents, and human–computer interaction require proper sarcasm detection to enhance the efficiency of such systems, since the detection error will have a negative impact on function. Prior research has demonstrated that sarcasm is not solely textual; it can be difficult to understand based on the text alone, and requires multiple modalities of evidence provided in text, audio and visual. Furthermore, the perception and understanding of sarcastic intent can vary significantly based on contextual and cultural factors (Dolare et al., 2024). Transformer-based models have also ushered in new developments in multimodal sarcasm detection, replacing traditional handcrafted feature engineering with deep-learning systems that can learn to detect cross-modal incongruity. The joint modeling of linguistic, prosodic and visual information has been found to be beneficial for sarcasm recognition, as evidenced by recent work on multimodal large language models, prompt-driven multimodal learning and multimodal contrastive learning (Li et al., 2025; Jana et al., 2025; Guo et al., 2026a; Guo et al., 2026b). In addition, optimization-based fusion approaches have enhanced the performance of multimodal systems by dynamically altering the fusion weight of the individual modalities, especially when facing noisy and limited data. The results underscore the need for adaptive fusion techniques for accurate sarcasm detection in practical conversational scenarios (Zhong et al., 2024; Payala and Jeet, 2026).

The detection of sarcasm is not an easy task despite its significance because there is a lot of ambiguity in the sarcasm expression. Sarcasm is frequently based on innuendo like facial expressions, tone of voice and body language, or inconsistency between the spoken word and intended interpretation. For example, a sentence such as “That was a great idea” may appear positive when interpreted from text alone; however, when accompanied by a sarcastic tone of voice or appropriate facial expressions, it conveys the opposite meaning. Background noise, differences in speaking style, and lighting conditions as well as diversity of the speaker are external factors that complicate the recognition of sarcasm. The conventional unimodal sarcasm detection methods are usually based on one modality of text, audio, or video. The text-based approaches emphasize lexical and contextual patterns without paying attention to vocal intonation and facial expressions. Audio-based techniques focus on prosodic and acoustic attributes (pitch, tone, energy) and are not able to understand linguistic context. In the same manner, the video-based methods examine the facial expression and gestures, and they might be ineffective in cases of occlusion, change in light, or low face visibility. Due to this fact, unimodal strategies tend to fail to effectively represent the subtle signals that are linked to sarcastic communication.

More up to date developments in DL and transformer architectures have added substantially to the ability of multimodal learning systems to learn more complex semantic and contextual connections. RoBERTa language models, being pretrained, have shown high efficacy in parsing deep contextual features in textual information. Equally, self-trained speech models like HuBERT can also be trained to learn expressive acoustic representations of subtle prosodic differences found in sarcastic speech. In the visual domain, video models that use transformers, like ViViT, are effective at capturing spatial and temporal dependencies in video sequences and, therefore, detect facial expressions and gestures that signify sarcastic behavior. Despite the ability of these models to give powerful modality-specific representations, a lot of existing multimodal sarcasm detection systems use a fixed combination rule that can only be used with fixed weights of each modality. These methods may not provide an adaptive tuning of contributions of modality depending on the quality or significance of the individual input streams.

To reduce these drawbacks, this study presents an optimization based multimodal sarcasm detection model that combines acoustic, visual and textual traits with advanced deep learning models. In particular, RoBERTa is applied to find textual information in context, dialog, based on the transcript, HuBERT is used to identify acoustic attributes related to sarcastic tone, and ViViT is used to learn spatiotemporal visual features of video sequences. In order to successfully concatenate such modality-specific representations, a Particle Swarm Optimization (PSO) based fusion approach is proposed to identify the best modality weights through minimizing classification loss. The resulting optimized feature representations are then presented to the two-layer fully connected classifier to get the final prediction of sarcasm.

The suggested framework re-formulates the multimodal fusion process as an optimization problem while existing multimodal sarcasm detection approaches rely on fixed multimodal fusion methods or attention-based multimodal fusion or graph-based multimodal interaction methods. The proposed Particle Swarm Optimization (PSO) based fusion strategy automatically optimizes the contribution of the textual, acoustic and visual representations by minimizing the validation classification loss during training without learning their modulation contribution or using a pre-defined set of rules. In such cases, this optimization-driven fusion kind of gives the framework authority to dynamically change the significance of the various modalities based upon their quality and discriminative capability which would boost robustness throughout noisy and incomplete multimodal situations. Moreover, the proposed framework is a model agnostic approach, where the fusion module can be attached to various pretrained encoders, without altering its internal architecture, facilitating a computationally efficient and easily transferable solution for other multimodal affective computing applications.

The suggested framework is evaluated using the public available MUStARD dataset which consists of textual, auditory and visual information for the sake of multimodal sarcasm detection. Based on the framework, the sarcasm detection mechanism is designed to be adaptive in order to improve the reliability and accuracy of sarcasm detection in various conversational contexts by optimizing the fusion. The following are a summary of the major contributions of this study:

1. An audio encoder based on HuBERT is used to derive acoustic and prosodic features representing tonal changes relating to sarcastic languages.
2. To recognize linguistic cues of sarcasm, the RoBERTa model is used to process textual transcripts & retrieve deep contextual representations.
3. Spatiotemporal visual features of video sequences are extracted with the help of ViViT architecture that captures facial expressions and gestures associated with sarcastic behavior.
4. An adaptive multimodal fusion strategy, based on Particle Swarm Optimization (PSO), is proposed, which can formulate the multimodal fusion as a global optimization problem, and solves the problem of whether to choose the fixed multimodal representation or manually set multimodal representation weights, automatically assigning the optimal multimodal representation weight.
5. The proposed optimization-driven fusion yields a framework that is more robust to noisy, ambiguous, or incomplete modalities when compared to the traditional attention-based and graph-based fusion methods, while keeping low complexity.
6. The complete architecture is tested on the MUStARD dataset in order to show the efficacy of the offered optimization-based multimodal sarcasm detection system.

By combining the development of advanced transformer structures and optimization-based multimodal fusion, the proposed system improves the performance of sarcasm detection and helps create more context-aware conversational AI and affective computers.

## 2 Related works

Gao et al. suggest an attention-driven fusion model to detect sarcasm incorporating information on speech, text, sentiment and emotion. The research points out the fact that the current multimodal solutions do not utilise the interactions between modalities, especially the interactions between sentiments and emotions. To address this, the authors combine text input received from the automatic speech recognition gesture with the sentiment analysis features, and combine emotion recognition with sentiment analysis. The results of the experiments on MUStARD++ show that it is effective to use emotional information and sentiment information together to achieve a high F1 score (Gao et al., 2024). Băroiu and Trăus -Matu examine the context detection of sarcasm and discuss the use of context data to understand sarcastic words. The authors test different DL models such as attention-based LSTM networks in the MUStARD dataset. As demonstrated by them, the attention mechanisms are particularly adequate to capture the context and to identify factors that make a dialog sarcastic. Another point discussed in the paper is how conversational history and speaker profiles can be used to enhance the performance (Băroiu and Trăușan-Matu, 2023).

Bhosale et al. (2023) concentrate on the multimodal sarcasm detection benchmarking & enhancement with the help of MUStARD and MUStARD++ datasets. The paper highlights that sarcasm is multimodal (text, speech, visual), and emphasizes the importance of sophisticated encoders for each of them. They present a trade-off on the drawbacks of the datasets and balanced extension of MUStARD which enhances the distribution of classes and improves model performance. Based on the experiment results, it is concluded that the present multimodal encoders and balanced datasets are efficient as reflected by an improvement in the scores of Macro-F1. Xi et al. introduce a new system of multimodal sarcasm detection named the Sentiment-Clue Inconsistency Global Detection Fusion Network (SCI-GDFN) to overcome the shortcomings of the current ones that mainly address the local inconsistency. The authors show that in many cases, sarcasm is caused by the contradictions created by the complexities between the textual and visual modalities that need to be analyzed globally and locally. To solve this, the framework suggests an active global capture module which simulates the space between each image-text pair. The results have been validated on some benchmark datasets such as MSD and Memotion and it shows good results in both datasets with high accuracy and F1 scores (Xi et al., 2025).

In this paper, Misra and Arora (2023) have introduced a relatively large-scale News Headlines Dataset that has high quality by mitigating major drawbacks of the existing datasets studied in-depth in this paper. Previously existing research in sarcasm detection either used large irregularly-labelled datasets (such as social media as gathered by using hashtags) or small high-quality labelled datasets by hand but of limited scale, which are unsuitable to thematically train deep learning models effectively. The authors solve this by generating a set of sarcastic headlines from The Onion and non-sarcastic headlines from HuffPost, and ensuring the labeling data was also reliable as well as the linguistic data. The authors propose a CNN-BiLSTM model architecture with attention mechanism to evaluate the data set, which is called Hybrid Neural Network. Local n-gram feature is given to the CNN, sequential feature to the BiLSTM layer, and attention layer to find important words used in the generation of sarcasm. The architecture diagram shows how to calculate the weights for the attention mechanism and how the weights are further combined with CNN features to get the final classification. As shown in the results table on page 4, the accuracy of the hybrid topology shown by the road experiments is 89.7 per cent which is higher than the baseline CNN model (84.88 per cent).

The study by Zambre introduces a deep learning architecture to identify sarcasm that is modular, which uses CNNs and context embedding models like BERT. The paper aims at capturing linguistic, emotional cues and contextual cues via a multi-layer architecture. Unlike traditional text properties, this framework incorporates sentiment analysis, context embeddings, and emotion detection to give a deeper understanding of the expression of sarcasm. The work has a merit in that it gives a comprehensive view of the issue of sarcasm detection and importance of multi-component architectures which model different aspects of language (Zambre and Bobade, 2025). Wei et al. (2024a) propose a global semantic awareness sarcasm detection model G2SAM that utilizes the graph-based representation of the multimode. The authors will say that the current graph-based models only capture the relationship between the individual instances, without capturing the global semantic relationships between the instances. To deal with this, G2SAM builds the fine-grained multimodal graphs per instance and maps them into a common semantic space. Moreover, the framework also uses graph contrastive learning utilizing labels to promote semantic consistency of representations. The current research will take a step toward enhancing the field by combining graph-based modeling with global semantic awareness in a more detailed multimodal sarcasm detection model such as the model proposed in the present work (Wei et al., 2024b).

Jin et al. pay attention to the global sarcasm detection through image-text fusion and present the feature reconstruction output algorithm for enhancement of interaction in two modalities. They use their mechanism attention as a way of learning low-rank representations of modalities features and the reconstruction of feature representations using weighted averaging. This reconstruction process helps in enhancing the quality of the features representation and ensures that the important information between the features is not lost. The work provides a contribution to the literature because it has shown that feature reconstruction and adaptive weighting can be effective in enhancing the detection of multimodal sarcasm, especially in image-text cases, where cross-modal inconsistencies are also very important sources of information (Jin et al., 2024).

For solving the problem of differential contribution of different modalities in multimodal fusion, Ma et al. (2024) considered two generative processes, and given a multimodal sarcasm detection framework. The standard ways of fuse promote the notion of equal significance of each modality, which can be biased. To overcome this, the proposed framework uses multimodal features and a dual generative process to make the model explore the emotional paradoxes in an efficient manner. Experimental results confirm that the approach achieves better results than existing practices, highlighting the need for modeling the contributions and inconsistencies across modalities. Experimental results display the superior performance of the approach over existing practices, and thereby underscore the need for modeling the contributions and inconsistencies across modalities. Bharti et al. propose a multimodal Sarcasm detector system which combines text and audio to overcome the limitations of unimodal systems. According to the paper, sarcasm is complex and, in most instances, it involves tonal and contextual cues which cannot be conveyed in text only. The classic models of text-based methods are unable to understand the tone, the emphasis and the speech patterns that are essential clues to sarcasm. To respond to this, the authors come up with a hybrid architecture which gets features on both modes of text and audio and fuses them into a coherent representation. The results reveal that multimodal fusion is more reflective approach to the user intent, particularly in conversation data (Bharti et al., 2022).

Hazarika et al. present a system called MISA (Modality-Invariant and -Specific Representations), moving the emphasis on multimodal sentiment analysis to the representation learning instead of fusion. According to the authors, the main issue with multimodal systems is the so-called modality gap due to the fact that the distribution of the text, audio and visual materials are not homogenous. To address this, MISA obtains two types of representations to each modality: modality-invariant and modality-specific. The invariant sub space aggregates common semantic knowledge across modalities by align the modalities in a common latent space that minimizes distributional gaps. Experiments using datasets data like MOSI and MOSEI, reveal that MISA outshines other state-of-the-art approaches greatly (Hazarika et al., 2020). Lin et al. introduce a hierarchical graph contrastive learning model (HGraph-CL) to consider intra modal and intermodal relationships of multimodal sentiment analysis. In many of the previous approaches, the emphasis was laid on the fusion of the features in different modalities, whereas this work emphasizes on the structural relationships within and across the modalities. To describe modality-specific relationships, the model draws unimodal graphs of each modality, e.g., language-based graphs of text, sequential graphs of visual and acoustic representations. They are then summed up into a multimodal graph which expresses interactions between modalities. To enhance the learning of the representation, the framework under consideration utilizes graph attention networks to put various weights on the nodes and relationships. The research is extremely valuable for research in the field of combining graph learning with contrastive goals to provide a more comprehensive understanding of the dynamics of multimodal sentiments (Lin et al., 2022).

They suggest a new version of the Multimodal Adaptation Gate (MAG) by Zhao et al., which is referred to as MAG+. First, M3R reinforces each modality with cross-modal attention, then it allows the model to learn the interaction and dependency between different modalities, and then fuse it. This is supplemented by a gating process which is a dynamic system of integrating multimodal information. The ALA module also improves the representation, by defining some features of various layers; both low level and high-level multimodal features are employed in the final representation. This paper extends the previous works by showing that adding attention processes to adaptive gating and hierarchical aggregation can achieve better and context adaptive multimodal sentiment analysis systems (Zhao et al., 2022). Tsai et al. present the architecture of Multimodal Transformer (MulT) that is created to address issues in multimodal language sequences, especially non-alignment and long-range modal dependencies. The problem can be solved by the directional pair-wise cross-modal attention, without explicit alignment, mulT. This way the model can learn to infer implicit relationships between data streams that do not occur at the same time, for instance a model that would know that the facial expression corresponds to a certain section of the text, even if they occur at different times. The architecture is constructed based on the common transformer architecture, and a few cross-modal attention blocks are added to enhance the representation multiple times via inter-modal interaction. It performs well on the benchmark tasks, such as the CMU MOSI, CMU-MOSEI and IEMOCAP, and it can outperform other previous state of the art techniques in both aligned and unaligned environments (Tsai et al., 2019).

Deng et al. study suggests the multimodal sentiment analysis framework, which is a cross-modal multi-head attention mechanism to surpass the limitations of the conventional fusion techniques. In this paper, it is emphasized that multimodal information, i.e., text, visual and possibly also audio information is at risk from both common and modality-specific information, and that sentences can be analysed thanks to these relationships. In the suggested model the information is first pre-processed & encoded in sequential forms with positional encoding so as to not lose any information about the semantic and contextual sequences in the system. Final classification is done using a feedforward neural network. Experiments conducted in the field of CMU MOSEI and MELD demonstrate that attention-based cross-modal fusion has higher accuracy and F1 scores (Deng et al., 2024). HuBERT suggests an unsupervised way of training the speech representations. It is trying to overcome such problems as the inability to know the sound unit and lack of segmentation using offline clustering (k-means) to obtain pseudo-labels. These labels and the learning of acoustic and language patterns are used to learn what words are covered by the mask and what is masked because of the mask, in a manner similar to a model like BERT. The more important issue is the regularity of the label, not that it is accurate. HuBERT performs comparably or better on benchmarks, such as LibriSpeech, with iterative clustering and large-scale training, surpassing models like wav2vec 2.0. Can achieve up to a 19% relative WER reduction. It is a scalable solution and can be applied in low resource settings as well as improve generalization to speech tasks (Hsu et al., 2021).

Contrary to the previous models of video classification that rely on CNNs, ViViT presents an all-transformer video classification model. It models the videos as sequences of spatio-temporal tokens, represented and manipulated by self-attention, enabling dependencies over a longer range. The model has been factorized to lower the high computational cost of the model by factorizing both spatial and temporal dimensions. It also employs regularization and trained image models to take the advantage of smaller datasets. A significant amount of state-of-the art benchmarks on Kinetics-400/600 and something-something v2 demonstrate that it significantly outperforms 3D CNNs. The article demonstrates design decisions in transformer-based video self-educates and how they can be scaled up and applied to the understanding of effective video tasks (Arnab et al., 2021).

To address the above challenges, Tian et al. (2023) introduced DynRT-Net, a multimodal sarcasm detection framework that uses dynamic routing to learn transformer modules adaptively based on the image-text input. In contrast to stationary models, it brings in dynamic routing to choose the transformer modules adaptively, with respect to the input pairs of image and text. The model is based on hierarchical co-attention approach to learn the most significant signal of sarcasm, cross-modal incongruity. It outperforms fixed architectures in the case of different levels of sarcasm (local and global inconsistencies). This is demonstrated by experiments being better than the state-of-the-art methods when used on benchmark datasets. The key contribution is the development of models of sarcasm detection as a dynamical process and not as a fixed one, which enhances greater flexibility to the multimodal inputs of diversity (Tian et al., 2023). The current paper complements multimodal detection of sarcasm by using gaze (eye tracking) characteristics. Gathering gaze is costly and based on this the authors project the synthetic gaze attributes and mix them with the actual ones. In practice, using the MUStARD++ data, they show that gaze patterns (fixations/regressions) can be employed to identify sarcasm. The model gets up to 6.6% performance increase with real gaze data and continues to gain with predicted data (even when the gaze is not present). It is the first “predict-and-use” in this area. This piece has shown the significance of cognitive cues in the interpretation of sarcasm in a conversational context (Tiwari et al., 2023).

The given paper suggests a semantic improvement strategy of multimodal sarcasm recognition. It contends that the current approaches have been placed undue emphasis on semantics of image-text discrepancy and fail to take into account semantics of image-text overall. The model learns multi-scale attributes of both textual and visual information and utilizes the contrastive learning to overcome the semantic gap among modalities. It also uses semantic similarities using samples in an attempt to enhance detection. Experiments show that up to 1,213 percent more accurate and F1-score than baseline. The point is that both inconsistency and understanding of the semantics are needed for accurate detection of sarcasm (Zhong et al., 2024). The multimodal, cross-lingual sarcasm detecting framework with text, visual, audio and cultural embeddings is discussed in this article. It deals with shortcomings of earlier models that do not consider cultural and linguistic differences. The system elicits a 12 per cent increase in accuracy with transformer-based architectures, multitask learning, and hierarchical attention toward data sets such as MUStARD and Memotion. The study emphasizes simulation of cross-modal dependencies and of culture. It provides a more comprehensive and generic approach to sarcasm detection in real-world applications, across words and domains of various languages (Dolare et al., 2024).

This paper requires a new activity, to make a sarcastic image and hashtags description. It produces a MuSG dataset with 5,000 samples and recommends a transformer to be used that fuses a visual feature, an OCR text and a hashtag. The model contextualizes multimodal incongruity so as to create human sarcasm. Uses joint embeddings and autoregressive decoding. There is high performance by both automatic and human evaluation. It is the first work on the topic of sarcasm generation (rather than its detection) in a multimodal context, and builds upon the existing research on tasks involving creative AI (Zhao et al., 2023). This paper compares the performance of LLMs and MLLMs in understanding sarcasm in written, audio and visual texts. The model evaluations are performed under the benchmarks (MUStARD++ (English), MCSD (Chinese)) and the model evaluations in zero-shot, few-shot and LoRA fine-tuning mode. It also investigates the use of LLMs as feature extractors as well as fusion mechanisms. The results suggest that cues like tone or prosody are highly informative when detecting sarcasm and multimodal cues (i.e., text + tone or prosody) are more effective than unimodal cues. It is possible to have MLLMs such as Qwen-Omni to perform competitively, with little or no supervision. The paper also emphasizes the significance of cross-modal reasoning and cross-lingual assessment, but also points out some shortcomings of current models to capture fined-grained pragmatic cues in conversational sarcasm in the real world (Li et al., 2025).

In this study, the authors present the novel framework, DMDP, of few-shot multimodal sarcasm detection under deep, modality-specific prompt tuning. The use of text and image encoders prompts is already being used in DMDP, rather than employing a single common prompt as in previous methods, and this is how it enables more specific capture of modality specific sarcasm indications. These cues are presented at multiple levels to make possible top-down learning of features. The model also includes a cross-modal prompt alignment and weighing, which takes into account different sarcasm conditions (text-driven, image-driven or incongruent). DMDP, thanks to the usage of pretrained models, such as CLIP, and loss reduction as prompts alone makes it effectively work in the low-resource worlds, where there is limited labelled data. Empirical results show good generalization to unseen data and show that power is steadily increasing over time compared to current baselines, a finding that suggests that deep prompt tuning can increase its power in the complex multimodal understanding tasks addressed. A strong model of multimodal sarcasm detection (MICL) is proposed in this paper, which is capable of solving the issue of spurious association in the current models. The authors highlight the fact that most of the models use too many “surface” textual or visual clues to trigger incongruity, rather than actual incongruity. To address this, MICL proposes multi view learning in three different perspectives: token patch (text image alignment), entity object relationships and sentiment level discrepancies. It uses contrastive learning and a fusion mechanism using a beta distribution to dynamically find out the importance of each perspective. Moreover, the model uses data augmentation of both text and imagery to enhance generalization and optimism toward any single modality. Tests on benchmark datasets indicate that MICL vastly outperforms state-of-the-art methods and is more resistant to confounding patterns, so that it is applicable in real-world sarcasm detection tasks (Guo et al., 2025).

To improve the interaction between multimodal and the semantic consistency, Xu et al. propose a framework of multimodal sentiment analysis called Hierarchical Correlation Modeling with Semantic Distribution Constraints (HCM-SDC) in this paper. Unlike the fusion methods which directly mix multi-modal features, the proposed method treats and models the intra-modal correlation and inter-modal correlation hierarchically, and introduces semantic distribution constraints to guarantee the compatibility between multi-modal feature representation. This will assist in eliminating inconsistencies in the semantic level and retain the discriminative information in model fusion. Experimental results are provided to show that there was an improvement in the performance of the benchmark multimodal sentiment analysis benchmarks over existing transformer-based and graph-based approaches, indicating that hierarchical correlation modeling for multimodal representation learning is effective (Xu et al., 2025).

But current graph-based models struggle to utilize the label information that can be beneficial to this multimodal sarcasm detection task. To overcome this, Wei et al. suggest a multimodal sarcasm detection method, Label-Aware Graph Contrastive Learning (LGCL). The approach proposed is to generate multimodal graphs from text and images, and employ label-aware graph contrastive learning to boost the semantic contrast between the sarcastic and non-sarcastic examples. Furthermore, back-translation augmentation is seamlessly applied to produce textual variation which queries the model with semantically equivalent text, resulting in higher model robustness and generalization. The evaluation experiment shows that the proposed framework can successfully fuse the three individual skills of graph reasoning, contrastive learning and data augmentation and achieve the best sarcasm detection effect. Experimental results demonstrate the feasibility of the suggested framework to integrate three skills mentioned above, graph reasoning, contrastive learning and data augmentation, and to provide the best sarcasm detection effect.

The authors of “A Multimodal Enhancing the Semantic Awareness Model for Sarcasm Recognition” propose a model called Enhancing Semantic Awareness Model (ESAM) for multimodal sarcasm recognition, which copes with the problem of “feature misalignment” and “noisy multimodal representation. Furthermore, it proposes a modality decoupled representation method and sentiment-based constraints to improve the semantic consistency between textual modality and the visual modality. Furthermore, there is an automatic outlier masking mechanism which identifies noisy samples, discards them from training, and enables the model to learn the informative multimodal representation. The experimental results demonstrate that the suggested usage of semantic-aware learning coupled with automatic outlier masking greatly outperforms the current semantic fusion frameworks (Yuan et al., 2025) in terms of accuracy and robustness of sarcasm detection.

In view of the reasoning power of the LLMs, Zhou et al. propose an LLMs Debate-Guided Network (LDGNet) for multimodal sarcasm detection. The framework suggests a multiple-LLM reasoning mechanism to assist in analysing inconsistencies in textual and visual information that is following the reasoning process in a debate. It enables the model to learn the complex semantic contradiction as well as the implicit sarcastic intent. The outcome of this experiment shows that SarcGd enhances the performance of sarcasm detection, and it provides better interpretability than traditional multimodal transformer-based methods (Zhou et al., 2025).

In this paper to overcome the restriction of existing approaches which use latent contextual encoding and multimodal fusion methods, Pan et al. put forward a cue-guided framework called CUCI-Net for multimodal understanding in conversation tasks to solve the problem of context-dependent conversational multimodal understanding. It is concluded that there cannot be a proper understanding of dependency between the dialog context and the dialog act without explicitly modeling the dependency between conversational utterances particularly in the case of sarcastic or ironic utterances. In turn, CUCI-Net suggests a 3-tiers model, which contains of Context-Utterance Structure Encoding, Global–Local Interpretation Cue Construction, and Interpretation-Cue-Guided Multimodal Interaction. The framework initially introduces structural differences between source and target, presenting local multimodal evidence from the text and global context information, and finally, the framework presents an explicit cue of interpretation to facilitate multimodal reasoning so that interaction across modalities can take place. The proposed framework yields superior results on both the proposed benchmark datasets, the MUStARD and the MUStARD++ compared to the rest of the state-of-the-art and has been shown to be an essential component for contextual reasoning in conversational multimodal understanding and sarcasm detection (Pan et al., 2026).

Although they fail to hold the deep level knowledge in the sarcastic content, multimodal sarcasm detection remains a challenge. To address this issue, Wei et al. suggest a knowledge-augmented graph reasoning framework to utilize knowledge graphs and enhance social signals. Conventional multimodal models fail to take into account common background knowledge or implicit user’s intention, which are crucial to the understanding of a text and image, each of them exhibiting sarcasm. To address this challenge, the framework proposes a Deep-level Knowledge Extraction Module based on large multi-modal models that can provide additional semantic knowledges from text-image pairs. Further, we present a Cross-Knowledge Graph Reasoning Module to obtain cross-knowledge graphs for deep-level knowledge to be correlated with the surface-level multimodal information, which is used for further semantic relation reasoning and sarcastic cue reasoning. Experiments with widely-used public multimodal sarcasm detection datasets confirm that the proposed framework outperforms current state-of-the-art techniques and demonstrates its effectiveness in integrating knowledge augmentation with graph reasoning to enhance multimodal sarcasm detection (Wei et al., 2025).

To address the unimodal shortcomings, Payala and Jeet (2026) introduced a multimodal DL framework for emotion recognition from combining textual, acoustic, and visual information. The conventional methods that use a single modality tend to have a noise sensitivity, lack of contextual knowledge, and poor generalization. To this end, the authors incorporate three of the most powerful models: DeBERTaV3 for extracting contextual features of the linguistic aspect of the text; derene for extracting acoustic and prosodic patterns in speech; and ResNet (2 + 1)D for extracting the spatio-temporal facial expression in a video (Zhong et al., 2024). One of the main contributions of the work is the implementation of an Aquila Optimizer (AO) to multimodal fusion. Instead of simply assigning a fixed or manually-tuned weight to each modality, AO learns a global weighting scheme that is applied dynamically, with minimal validation loss. This allows the system to put importance on the most informative modality given various conditions that is more robust to the absence or noisiness of one of the modalities. The combined results of modality outputs are weighted to come up with the final prediction (Zhong et al., 2024). The model is tested on two datasets of real-life data, CREMA-D and IEMOCAP, which include six emotions, anger, disgust, fear, happiness, neutral, sadness. This has been shown to yield better results in accuracy and generalization than unimodal and traditional methods, especially using fusion methods. The results of the explainability analysis of SHAP indicate that all audio features, followed by video features, and text features are predominant in affecting the model, with the latter being complementary across emotion types (Payala and Jeet, 2026).

The reliability of any single modality and how the lack of observation affects the reliability have also received increased attention in recent multimodal sarcasm detection research. When one or more modalities are noisy or unavailable, reliability-aware multi-view fusion approaches aim to adjust the contribution of each modality in order to take its reliability into consideration. It is also quite relevant for conversational sarcasm, as text, voice and image cannot be equally effective for each sentence. The need to explicitly model the reliability of modality is made evident by such approaches that assume that not all modality evidence will be equally informative.

A more recent line of research involves refining the complementary encoders and the multilevel crossmodal interaction. These methods seek to enhance multimodal representations by explicitly capturing the relationship between modalities at several representation levels instead of just composing a set of modality-specific features (Guo et al., 2026b). These approaches help to emphasize that the multimodal system has to be more than the sum of its parts and that the exchange of complementary information between the modalities is vital. The present study, however, emphasizes optimizing the contribution of already extracted textual, acoustic and visual representations by using a global modality-weight optimization mechanism.

In recent years, other research on CLIP-based and LLM multi-modal sarcasm understanding has been conducted as well. In Interactive CLIP, every task in each domain is conditioned on the outputs of the other, with the aim of revealing the potential of pre-trained vision-language representations for learning multimodal semantic relationships studied in InterCLIP-MEP (Chen et al., 2025). Other recent studies involve using large-scale vision-language models for sarcasm detection, where models have been shown to be capable of reasoning over visual and linguistic information (Chen et al., 2025). They mostly rely on large-scale multimodal representation learning and reasoning, while the proposed framework relies on the dedicated multimodal encoders of text, speech and video, and examines the optimization of the feature-level contribution to multimodal reasoning.

Multimodal systems also need to be assessed in the context of complex affective and semantic interpretation environments, such as benchmark-oriented research (MER-Bench) (Nie et al., 2026). The studies conducted in these recent works highlight a gradual shift from traditional feature concatenation to reliability-aware fusion, multilevel crossmodal interaction, CLIP-based modeling, and large vision-language model reasoning. These methods are however different from the current work with their initial modeling objectives. The proposed framework explores a compact optimization-based fusion approach, where Particle Swarm Optimization is used to look for a best-found global modality-weight configuration based on textual, acoustic and visual representations. During this fusion search, the pretrained modality encoders do not change, and thus the contribution of the fusion mechanism can be isolated using controlled ablation experiments.

## 3 Proposed methodology

This study analyzes sarcastic and non-sarcastic expressions in various modalities using MUStARD dataset. MUStARD is a multimodal sarcasm detection dataset benchmark which consists of aligned audiovisual clips and respective textual transcripts, allowing the multimodal analysis to be performed comprehensively. The data was in the form of conversational video clips of television sitcoms, and the annotation of sarcasm on each utterance is done as either sarcastic or non-sarcastic.

One of the most difficult aspects of sarcasm recognition is that sarcastic intent is seldom expressed via a single modality. Sarcasm has been represented using several cue systems but is most frequently conveyed with prosodic variations, facial expressions, temporal gestures and/or textual-grammatical and/or semantic context. As a result, it is usual to encounter ambiguous predictions using just one modality. A framework based on wireless multi-modal communication, consisting of dedicated encoders for different communication modalities is proposed. RoBERTa is used to extract contexts from the conversation in the transcripts and extract semantic inconsistencies in these contexts, HuBERT is used to learn subtle acoustic features like pitch, stress and speaking style, which are often satiating sarcasm in the transcript, and ViViT is used to model spatiotemporal expressions and gestures in the face that are hard to observe by just viewing facial images. These complementary representations are then fused adaptively to harness the cross-modal information for robust sarcasm detection by using the proposed PSO-based adaptive fusion strategy. The visual modality is presented as video clips (usually in the.mp4 format), whereas the audio one can be presented as speech signals which can be further treated as.wav files to extract acoustic features. Moreover, the speech is accompanied with the related textual transcript, which records the linguistic data of a conversation. There is also contextual dialog before the target utterance that is especially important in discerning sarcastic intent as sarcasm can be considerably reliant on conversation context. The MUStARD dataset was sampled based on the popular TV series like Friends, The Big Bang Theory, and The Golden Girls where sarcastic expressions appear very often in natural dialog. Standardized preprocessing of the video, audio, and textual data is used to guarantee uniform processing of the modalities before extraction of features. These preprocessing processes help in efficient training and testing of the suggested multimodal sarcasm detection scheme. The general workflow of the proposed architecture, such as modality-specific encoders and the Particle Swarm Optimization (PSO) based fusion mechanism, is shown in Figure 1.

**Figure 1:**
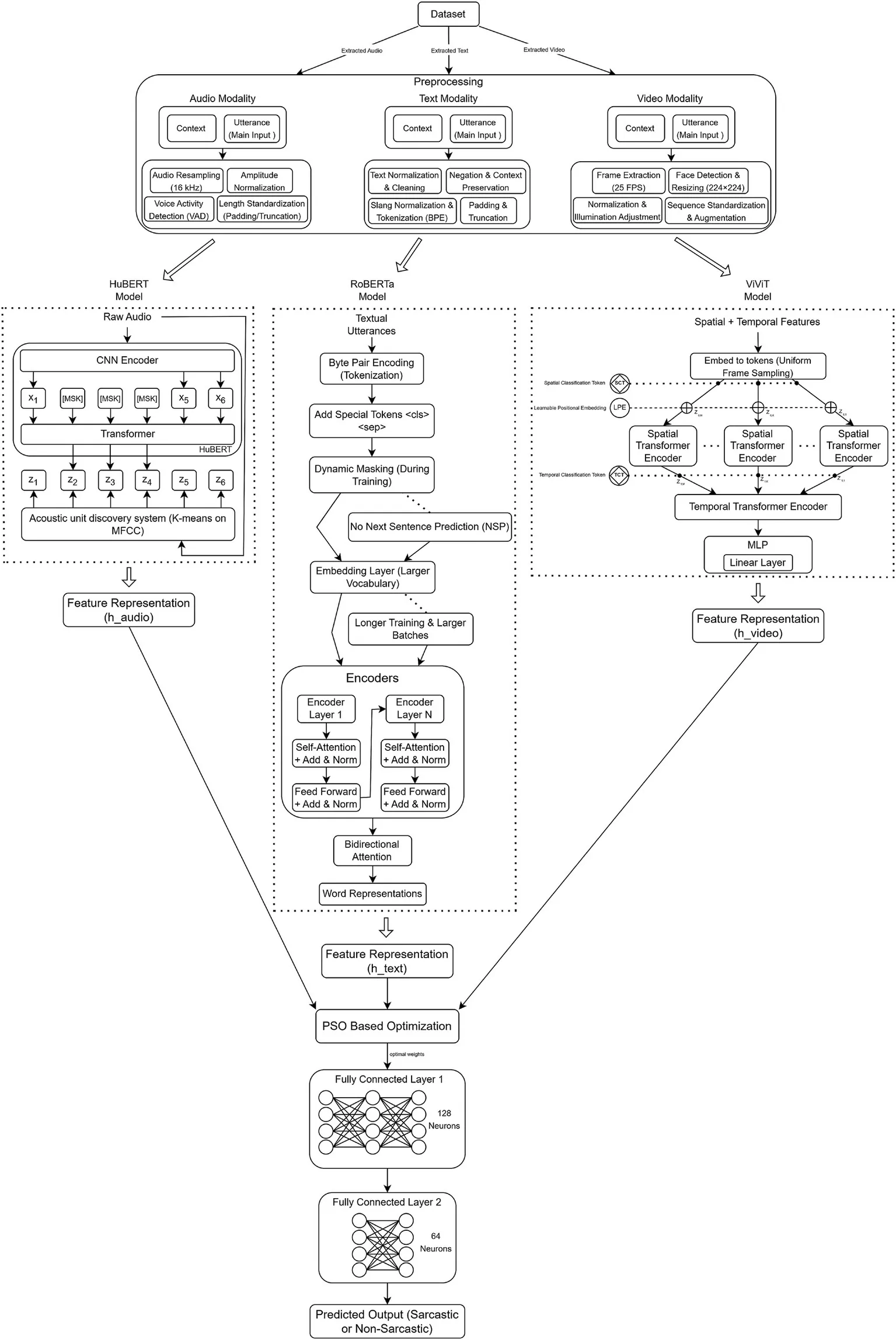
Structural design of the proposed multimodal sarcasm detection approach.

Acoustic information is of significance importance for sarcasm detection due to the fact that sarcastic expressions are often marked by giving the impression of a change of pitch, of stress, of speaking rate, of pauses and of intonation, which cannot be captured by a textual transcription. The temporal relationships like this are not well represented by the conventional handcrafted acoustics. HuBERT is therefore chosen due to its self-supervised transformer model learned representations of speech, able to capture subtle prosodic variations that have strong correlations with sarcastic speech.

Speech signals were also recorded in the audio modality, the video clips of the MUStARD dataset were converted to equal processing of all the samples, to.wav format of the waveform files. To fit the HuBERT architecture audio input format (16 kHz), the audio recordings were resampled, and amplitude normalization was used to minimize differences due to recording conditions or speaker loudness. It used a Voice Activity Detector (VAD) to detect and delete non-speech intervals, therefore, excluding silence, noise in the background, and non-relevant pauses, but only keeping active speech that can be used as a marker of sarcasm. Speech signal and background noise were also preprocessed using spectral filtering to improve the clarity of speech signal. Because the length of utterances in the various video clips was not the same, all the audio samples were equalized to a constant temporal length, by truncating longer sample and zero padding shorter samples.

### 3.1 Audio modality for HuBERT model

This gave uniform dimensionality and time correspondence of all inputs when training. These preprocessing operations improved the quality of the speech signals and allowed the HuBERT model to extract meaningful acoustic representations that reflect subtle prosodic and tonal features of speech related to sarcasm. The HuBERT structure that was adopted to extract acoustic features in the audio is shown in Figure 2.

**Figure 2:**
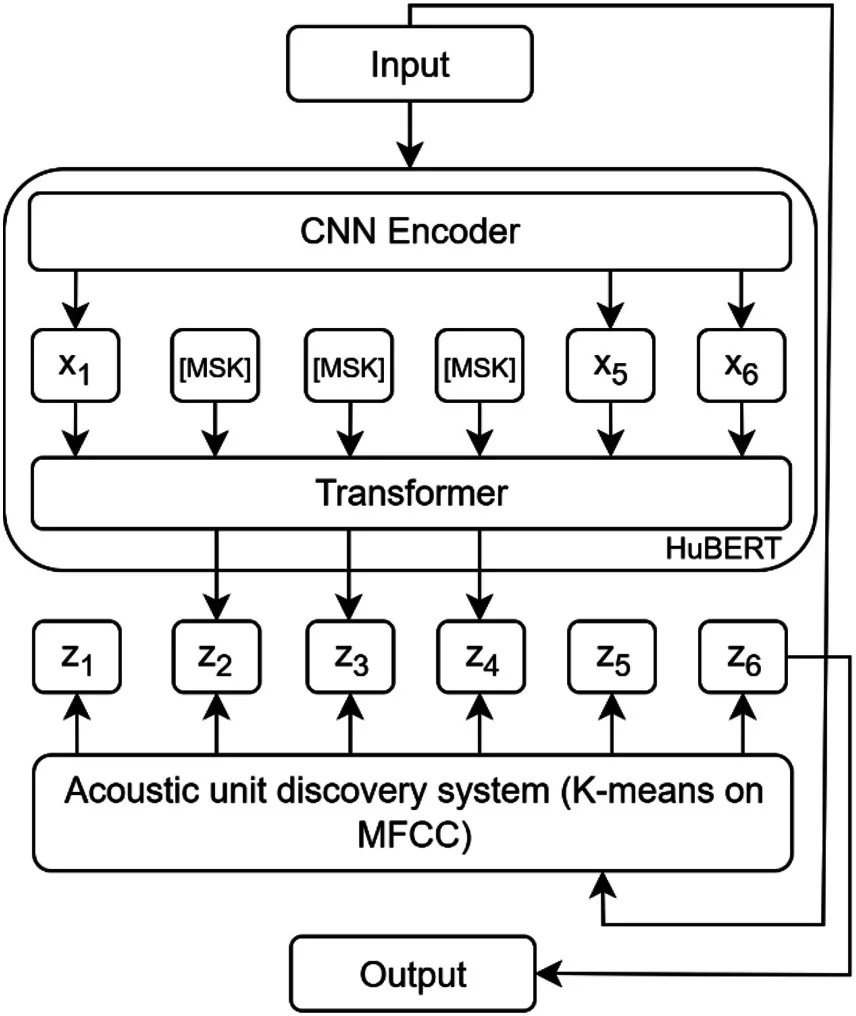
HuBERT-based architecture for extracting audio features.

#### 3.1.1 Input audio representation

The input audio waveform is given by Equation 1:

$$a=\{a_{1},a_{2},\ldots ,a_{L}\}$$ (1)

In which *a_i_* is the amplitude of the signal at sample number *i* and *L* is the number of samples in the waveform.

Audio input is further separated into two different parts namely context audio and utterance audio. The context audio is the previous dialog/history of conversation and gives some situational information, and the utterance audio is the segment of the speech that is the target of the sarcastic utterance that is to be categorized.

Context and utterance are analyzed independently as two audio waveforms, and the model, according to Equation 1, is able to identify temporal and prosodic changes in each segment. The separation empowers the model to acquire associations between the previous conversational tone (context) and the pattern of the present spoken expression (utterance) which is essential to the accurate sensing of sarcasm in multimodal contexts.

#### 3.1.2 Convolutional feature encoder

The raw waveform is given to the HuBERT convolutional feature encoder where the raw signal is mapped to latent frame-level acoustic features. The process can be given in Equation 2 as:

$$h_{a}=f_{\text{conv}}(a)$$ (2)

Where *f_conv_(·)* refers to the convolutional feature encoder of HuBERT and *h_a_ ∈ R^T′ × d^* is the latent speech representations that have been extracted where *T′* is the encoded frame count and *d* is the feature dimension.

#### 3.1.3 Transformer contextual encoder

The speech representations are encoded and then fed through a contextual encoder, which is a Transformer based encoder with multiple layers of self-attention. The module is a representation of long-range dependencies and contextual acoustic patterns in the speech sequence as shown in Equation 3:

$$c_{a}=f_{\text{transformer}}(h_{a})$$ (3)

And *f_transformer_(·)* is the stacked Transformer encoder layers, *c_a_ ∈ R^T′ × d^* represents the contextualized acoustic representation.

#### 3.1.4 Layer freezing and fine-tuning

A pretrained HuBERT model is utilized, so the lower layers involved in the learning of general acoustic representations are frozen, and the upper Transformer layers are fine-tuned on the MUStARD dataset. This process can be formulated in Equation 4:

$$c_{a}^{*}=f_{\text{train}}(c_{a}^{\left(u\right)})$$ (4)

*c_a_^(u)^* is the output of the unfrozen Transformer layers, and *f_train_(.)* indicates the fine-tuned parameters that are optimized on sarcasm detection.

#### 3.1.5 Temporal pooling

A pooling operation is performed to get a fixed length utterance representation of the contextual sequence shown in Equation 5:

$$\bar{c}=\text{Pool}(c_{a}^{*})$$ (5)

Where *c̄_a_ ∈ R^d^* is the summary of audio embedding at the global acoustic level of the utterance.

#### 3.1.6 Classification layer

The embedded pool is then projected to the sarcasm classification space by a linear transformation shown in Equation 6:

$$Z_{\text{audio}}=W_{\text{audio}}c_{a}^{-}+b_{\text{audio}}$$ (6)

Where *W_audio_* and *b_audio_* are parameters that are trainable.

#### 3.1.7 Sarcasm probability prediction

Lastly, a softmax activation function is implemented to get the probability of sarcasm classes as in Equation 7:

$$p_{\text{audio}}=\text{softmax}(Z_{\text{audio}})$$ (7)

Such that *p_audio_ ∈ R^C^* and C = 2 is a category of sarcasm (sarcastic, non-sarcastic).

The resultant probability vector *p_audio_* records changes in pitch, tone, rhythm, acoustic cues of sarcastic speech. The audio predictions are then merged with the textual and visual modality predictions at the multimodal fusion stage.

The HuBERT-Base model that has been trained on the LibriSpeech dataset was finetuned on the classification of sarcasm with the help of the PyTorch framework and the HuggingFace Transformers library. In this model, 768-dimensional contextual embeddings were produced and the mean pooling was used to get an utterance representation of fixed length. To avoid overfitting a dropout layer (rate = 0.3) was used before the final classification layer.

The AdamW optimizer was used with a learning rate of 1×10^−4^, weight decay of 1×10^−5^, and a batch size of 32 to train the model. To avoid overfitting, early stopping with patience = 5 based on the validation loss was applied.

Mixed precision (FP16) was used to train the maximum number of epochs (30) to optimize the use of GPUs. The warm-up was linear up to 10% of training, and cosine decay for the remaining training steps. Gradient clipping (max norm = 1.0) was used to stabilize training. All the audios fed into the inputs were resampled to 16 kHz and shortened to the same length to ensure uniformity in the processing of the batches.

### 3.2 Video modality for ViViT model

Facial expression, gestures (eyes, head and body) and body movement are important cues in sarcastic intent, especially in cases where text and acoustic cues are ambiguous. The use of sarcasm is not static during a single sentence, so this requires modeling of timing in facial movements. Hence, the ViViT architecture is used that incorporates both spatial and temporal dependency of data with the help of self-attention mechanism in the Transformer.

The video clips in the MUStARD dataset were broken down into sequence of frames in the video modality to maintain the temporal continuity of visual expressions. The videos were cut into frames at 25 frames per second (FPS) so that the time-related data would remain the same. Each frame had the facial part of the speaker identified by a deep-learning based face detection algorithm, which enabled the model to concentrate on the facial expression and body movements related to sarcastic communication. The identified face areas were cut and scaled to 224×224 pixels, the size of the input to the ViViT architecture. Histogram equalization was used to minimize the effect of differences in illumination among recordings and pixel values were converted to the range [0,1] to stabilize the training process.

The extracted frames were combined into the fixed video clips to get the dynamics in space and time. Repeated frames were used to fill in the shorter clips and evenly cut the longer clips to make them the same length. To make the models more robust to varying recording conditions, data augmentation techniques like random horizontal flipping, rotations and brightness adjustment were applied. These pre-processing steps have helped the ViViT model to learn meaningful spatio-temporal patterns of facial expressions that capture the context-specific nuances of sarcasm, as well as gestures. The entire structure of the ViViT model that was used to learn the representation of spatio-temporal video is shown in Figure 3.

**Figure 3:**
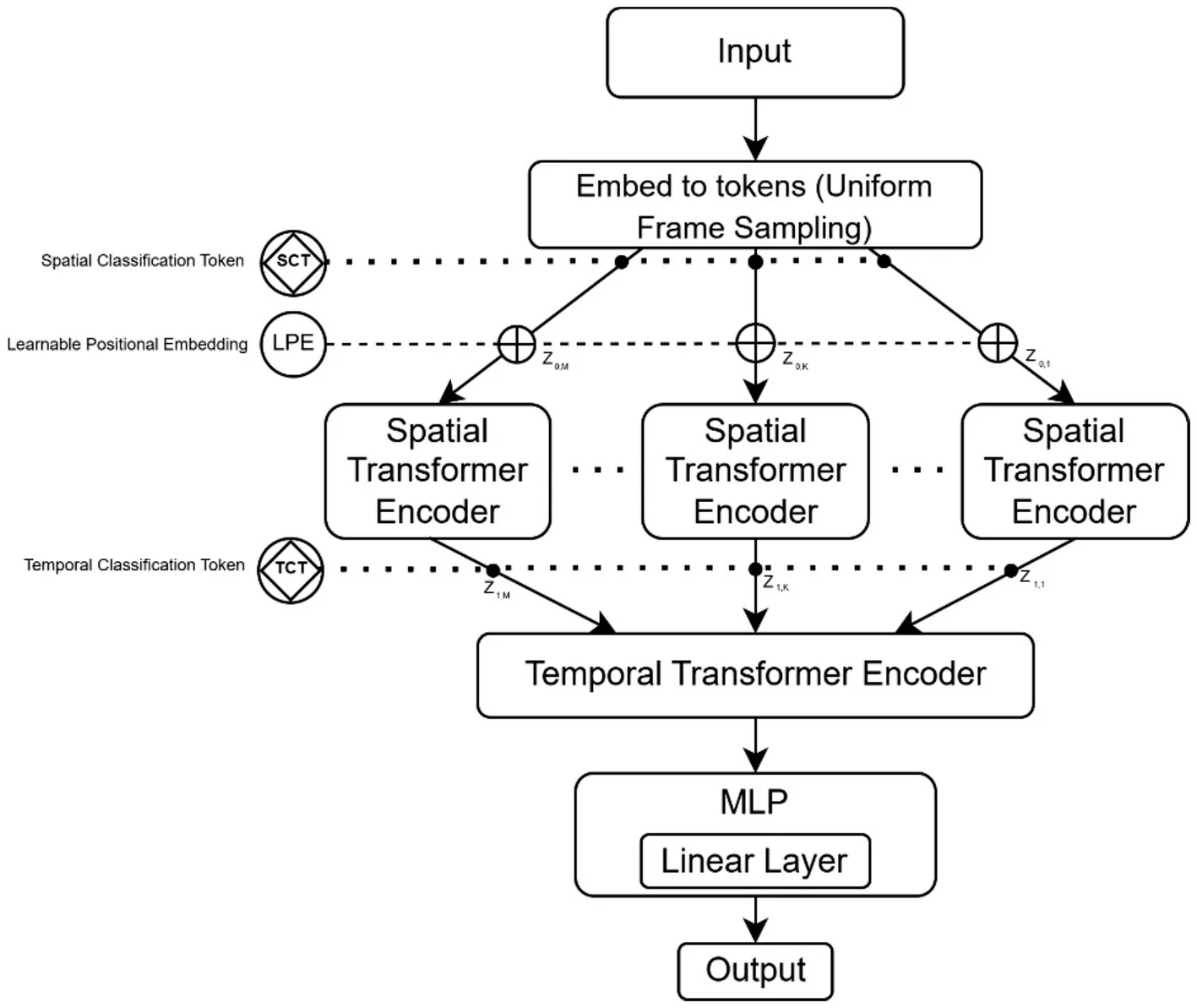
Architecture of the ViViT model for spatio-temporal video modeling.

#### 3.2.1 Video input representation

The input video sequence is a sequence of frames and is represented by Equation 8:

$$V=\{v_{1},v_{2},\ldots ,v_{T}\}$$ (8)

Where *v_t_* is the *t^th^* video frame and *T* is the number of video frames in the clip.

The video input is separated into two separate parts, namely, context video and utterance video. The context video records the context of the previous visual picture and facial expression that offer contextual and emotional clues whereas the utterance video aligns with the target part, which comprises of what the speaker says when making the sarcastic statement.

Both the context and utterance videos are followed as individual frame sequences by Equation 8. These sequences are then sent to the ViViT model that encodes the spatio-temporal features of each segment. This division allows the model to acquire the dependency of previous visual data and the current facial and gestural data which are essential in correct sarcasm recognition in multimodal analysis.

#### 3.2.2 Patch embedding

Every frame is broken down into non-overlapping spatial patches and encoded into embedding vectors. This turns the video frames into a stream of patch tokens which can be processed by the transformer as shown in Equation 9:

$$x_{p,t}=f_{\text{patch}}(v_{t})$$ (9)

And *f_patch_(.)* is the patch embedding operation and *x_p,t_* is the embedding vector of the *p^th^* patch on frame *t*.

The input sequence of tokens is the set of patch embeddings of all frames and is shown in Equation 10:

$$X=\{x_{1,1},x_{2,1},\ldots ,x_{P,T}\}$$ (10)

Where *P* is the number of spatial patches per frame.

#### 3.2.3 Spatio-temporal transformer encoding

The patch tokens are fed through a multi-head self-attention layers Transformer encoder, to both get the spatial relationships in frames and temporal dependencies across frames. This process is represented in Equation 11:

$$H=f_{\text{ViViT}}(X)$$ (11)

With *f_ViViT_(.)* indicating the stacked Transformer encoder levels of the ViViT design and *H ∈ R^N × d^* indicating the contextualized spatio-temporal token representations.

#### 3.2.4 Video representation aggregation

A pooling operation is used over the token embeddings to get one representation of the whole video clip shown in Equation 12:

$$\hbar_{v}=\text{Pool}(H)$$ (12)

With *ħ_v_ ∈ R^d^* the aggregate video embedding of global spatio-temporal facial patterns.

#### 3.2.5 Classification layer

The video representation is summed up to an aggregated video representation projected into the sarcasm classification space using a linear transformation as shown in Equation 13:

$$Z_{\text{video}}=W_{\text{video}}\;\hbar_{v}+b_{\text{video}}$$ (13)

Where *W_video_* and *b_video_* are parameters that are trainable.

#### 3.2.6 Sarcasm probability prediction

The softmax function is used to obtain the final probability distribution over the sarcasm classes shown in Equation 14:

$$p_{\text{video}}=\text{softmax}(Z_{\text{video}})$$ (14)

Such that *p_video_ ∈ R^C^* and C = 2 is the categories of sarcasm (sarcastic and non-sarcastic).

Visual cues that can be used to determine the sarcastic intent include facial expressions, gestures, and some temporal variations that are captured by the probability vector *p_video_*. These predictions are subsequently fused with audio and textual modality output at the multimodal fusion phase.

This research entails fine-tuning the ViViT-Base model which was initially trained with huge video data sets to the sarcasm detection problem using PyTorch and HuggingFace Transformers. The input clips were sampled at 16 frames taken at 25 frames per second and resized to 224 × 224 in resolution. The pixel values were scaled to [0,1] range and data augmentation methods such as random horizontal flipping and variation in brightness were used so that the model can generalize better.

The final classification layer was modified to change the classification for sarcasm to binary classification. It used AdamW optimizer with learning rate 1×10^−4^, batch size 16 and weight decay 1×10^−5^. To minimize overfitting, there was a dropout layer with rate = 0.3 placed before the classifier. Early stopping (patience = 5) was also used, with a maximum of 30 epochs trained, depending on the Validation performance. A linear warm-up cosine learning rate decay schedule was employed to make sure that the convergence was stable during training.

### 3.3 Text modality for RoBERTa model

The fact that when someone is saying something with sarcasm, there will usually be a semantic incongruity, contextual contradiction, exaggeration or implication throughout the linguistic context. The contextual dependencies, or histories of conversations, are difficult to capture with traditional word embedding techniques. Due to its strong bidirectional contextual representation learning ability, RoBERTa is thus used, as it can capture the subtle semantic relationship and inconsistency information that sarcastic dialog presents.

The textual data preprocessing was made in a way that would not destroy linguistic patterns that might be used to signify sarcastic intent but be compatible with transformer-based models like RoBERTa. Standard normalization steps (lowercasing, punctuations, and tokenizing) were incorporated into the preprocessing pipeline, but contextual and semantic information that is critical to detecting sarcasm was preserved. In contrast to aggressive methods of removing stop-words, a selective filtering method was followed in order to retain those words that add to a sarcastic interpretation. Emphasis modifiers (e.g., very, extremely), intensifiers and context cues were not eliminated because they frequently add to the sarcastic tone. Likewise, the words of negation like not, never or short forms like do not or cannot were also retained since negation can completely change the intended meaning of a statement.

In order to manage conversational variability that appeared in the MUStARD transcripts, informal expressions and lexical slang were normalized with a lexical mapping rule and then tokenized. Informal constructions like gonna or wanna were translated into more standard constructions (going to, want to) to diminish the lexical sparsity keeping the semantic meaning. Word prolongations through emphasis (e.g., soooo good) were vetted with caution to avoid loss in expressiveness. The RoBERTa tokenizer consists of the subword tokenization of the word by the method of the so-called Byte Pair Encoding (BPE), where rare or unseen words are further broken down into smaller units that are meaningful and help the model to address the question of vocabulary differences.

Truncation and padding were used for making sure that length of all input sequences were same to batch process. Care was also exercised not to erase sentence endings or other contextual cues that could have sarcastic implications. The textual modality is supported through this preprocessing pipeline so that the textual modality can be sensitive to minor linguistic details like contrast in context, exaggeration, negation, and pragmatic cues that can be related to sarcasm. The RoBERTa network architecture employed in the extraction of contextual textual features is shown in Figure 4.

**Figure 4:**
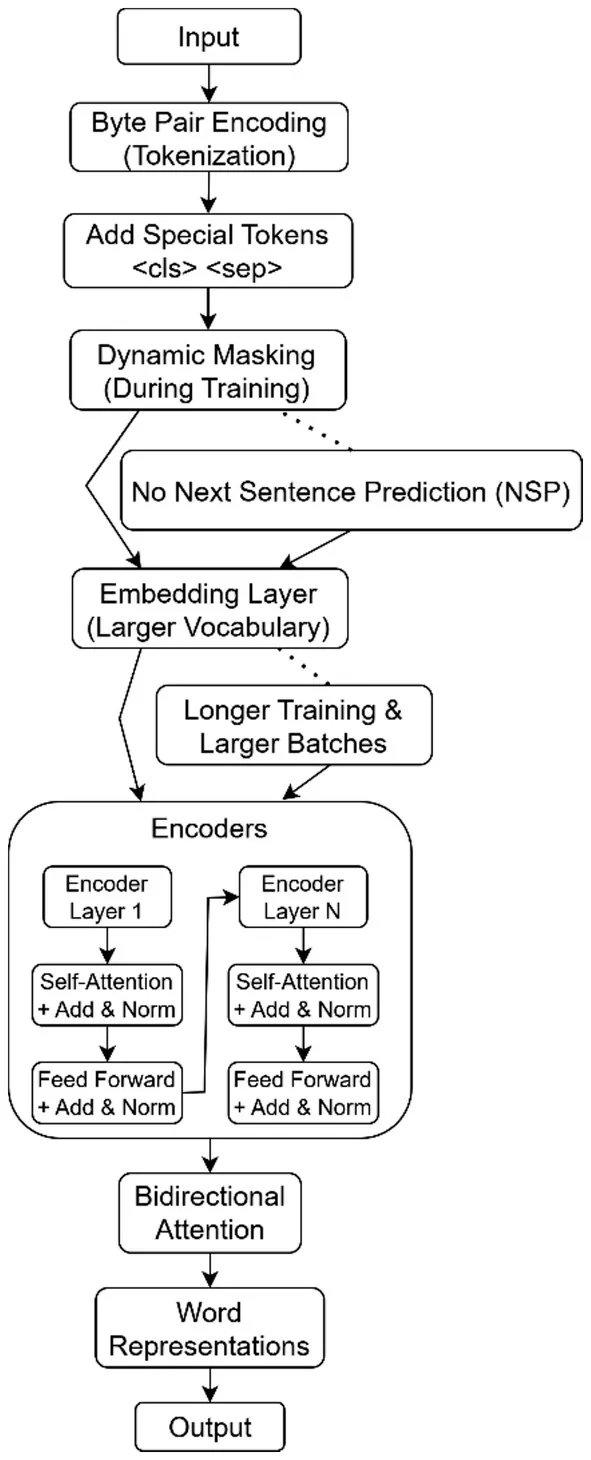
RoBERTa-based architecture for contextual representation of text.

#### 3.3.1 Text input representation

The input transcript represents a sequence of tokens as stated in Equation 15:

$$x=\{x_{1},x_{2},\ldots ,x_{T}\}$$ (15)

Where *x_i_* is the *i^th^* token in the transcript and *T* is the length of the sequence.

The input text is made up of context and utterance, which are joined in a single sequence and then processed. The context text contains pre-conversation information, whereas the utterance text is related to the target sentence the sarcasm of which must be recognized.

The context and utterance tokens are added together with the help of special tokens that separate them; the interdependencies between context and utterance are then learned by the model. This concatenated expression is then inputted into the RoBERTa model, which encodes contextual relation and semantic subtleties on the whole sequence. The model can use this approach to use both previous conversation and the target utterance to achieve better performance on sarcasm detection.

#### 3.3.2 Token embedding

The token sequence is transformed into a dense form and can be presented as a vector with the help of the RoBERTa embedding layer; token representations are combined with positional representations as shown in Equation 16:

$$h_{0}=\text{Embed}(x)$$ (16)

Where *h_0_ ∈ R^T × d^* is the embedded token sequence, and *d* is the embedding dimension.

#### 3.3.3 Transformer-based encoding mechanism

Several transformer layers then consume the token representations that apply multi-head self-attention to detect contextual dependencies between tokens. The contextual representation can be calculated in Equation 17 as:

$$H=f_{\text{RoBERTa}}(h_{0})$$ (17)

Where *f_RoBERTa_(·)* is the sequence of stacked Transformer encoder layers of the RoBERTa model and *H ∈ R^T × d^* is the contextualized token representations.

The self-attention mechanism used in RoBERTa computes attention weights between tokens using the following formulation shown in Equation 18:

$$\text{Attention}(Q,K,V)=\text{softmax}(\frac{QK^{T}}{\sqrt{d_{k}}})V$$ (18)

In which *Q*, *K*, and *V* are matrices of query, key, and value, respectively, based on the token embeddings, and *d_k_* is the dimension of the key vectors.

#### 3.3.4 Sequence representation

The final Transformer layer is used to extract the contextualized embedding of the special classification token [CLS] to receive one representation of the entire sentence as shown in Equation 19:

$$h_{\mathrm{CLS}}=H_{\left[\mathrm{CLS}\right]}$$ (19)

Where *h_CLS_ ∈ R^d^* is the collective representation of the whole text sequence.

#### 3.3.5 Classification layer

The resultant sentence representation is then linearly transformed into the sarcasm classification space as shown in Equation 20:

$$Z_{\text{text}}=W_{\text{text}}h_{\mathrm{CLS}}+b_{\text{text}}$$ (20)

Where *W_text_* and *b_text_* are trainable parameters.

#### 3.3.6 Sarcasm probability prediction

The probability distribution of the classes of sarcasm is determined with the help of the softmax activation function as shown in Equation 21:

$$p_{\text{text}}=\text{softmax}(Z_{\text{text}})$$ (21)

Where *p_text_ ∈ R^C^* and C = 2 are the sarcasm categories (sarcastic and non-sarcastic).

Output *p_text_* is the prediction vector of the textual modality, i.e., the contextual linguistic indicators that can be a hint of sarcastic content. Outputs of the audio and video modalities are then later fused with these predictions in the multimodal fusion step.

The pretrained model, A RoBERTa-Base, was fine-tuned on the sarcasm classification task on the PyTorch framework and HuggingFace Transformers library. A max sequence length of 128 tokens was applied and the official RoBERTa tokenizer was used. Short sentences were padded and longer sentences were cut without excessive use of a sentence.

The utterance representation was taken to be the embedding of the [CLS] token of the last Transformer layer. To fine-tune the model, the AdamW optimizer with a learning rate of 2×10^−5^, batch size of 32, weight decay of 1×10^−5^ and dropout of 0.3 was used in the classification head. The number of epochs trained was capped at 30 epochs and early stopping (patience = 5) was performed using the validation performance.

Linear warming up done first for the first 10 percent of training steps and cosine learning rate decay after. Gradient clipping (max norm = 1.0) was used to stabilize the training process and gradient explosion was avoided.

### 3.4 Multimodal fusion with particle swarm optimization

Even though unimodal streams of information (video, audio and text) can be used to obtain useful cues pertaining to sarcastic expression, using a single modality can also result in unreliable predictions in cases where the input signal is noisy, incomplete, or ambiguous. As an illustration, textual transcripts can be devoid of tonal details, audio cues can be overwhelmed by peripheral noise and visual cues can be influenced by an occlusion or a change in illumination. In order to overcome these constraints, a multimodal fusion mechanism is embraced in order to synthesize complimentary information of all modalities. Rather than applying a predetermined or fixed fusion strategy, the piece utilizes an optimization-based method of fusion with the help of Particle Swarm Optimization (PSO) to decide on the best contribution of each modality.

The choice of modality specific encoders is not enough to address multimodal sarcasm detection since the modality-specific information is different based on the conversation context. For instance, some sneering remarks may be mainly dependent on intonation of the voice while others may be more dependent on the contextual language and/or expressions in the face. Failure to give equal weight to each modality will therefore lead to lower classification accuracy. This is the main reason for proposing the adaptive fusion method using PSO that automatically allocates the best fusion weight of each modality based on its discriminative ability.

The general structure of the multimodal fusion system, PSO module and the ultimate classification layers are shown in Figure 5.

**Figure 5:**
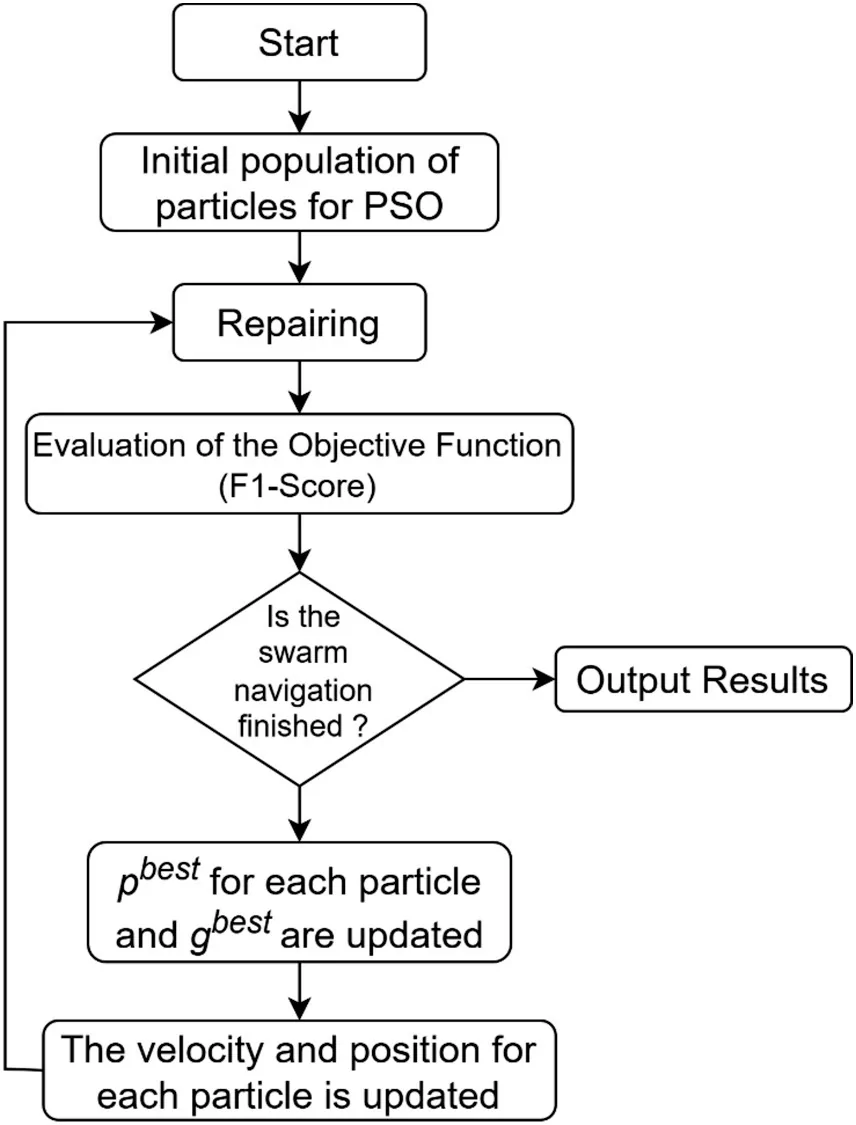
PSO-enabled architecture for multimodal feature fusion.

Denote the feature representations derived in the text, audio, and video encoders as shown in Equation 22:

$$h_{\text{text}},h_{\text{audio}},h_{\text{video}}\in R^{d}$$ (22)

In which *h_text_*, *h_audio_*, and *h_video_* represent the feature embeddings of the RoBERTa, HuBERT and ViViT models respectively, and *d* is the embedding dimension. These modality-specific features are taken out of the pre-trained models following unimodal training and are being used as inputs into the optimization phase.

#### 3.4.1 PSO-driven optimization of modality weights

PSO is employed to find the most suitable weight distribution between modalities. Every member of the swarm is a candidate weight vector that is defined as shown in Equation 23:

$$a^{\left(0\right)}=\{a_{\text{text}},a_{\text{audio}},a_{\text{video}}\}$$ (23)

Subject to the constraints:

$$\sum_{\mathrm{m\epsilon M}}a_{m}=1,a_{m}\geq 0$$

Where *M = {text, audio, video}* represents the set of modalities.

In the optimization process, the particles are updating their position and their velocity using their personal best solution and the best solution globally that is found by the swarm. The rule of velocity update is given in Equation 24:

$$v_{i}^{t+1}=wv_{i}^{t}+c_{1}r_{1}(p_{i}-x_{i}^{t})+c_{2}r_{2}(g-x_{i}^{t})$$ (24)

Where

- *v_i_^t^* indicates the speed of the particle *i* at iteration *t*.
- *x_i_^t^* is the current weight vector.
- *p_i_* is the most known position of the particle.
- *g* is the optimal solution in the world.
- *w* is the weight of inertia.
- *c1 and c2:* acceleration coefficient.
- *r_1_, r_2_ ∈ [0,1]* are random numbers.

The particle position is updated as shown in Equation 25:

$$x_{i}^{t+1}=x_{i}^{t}+v_{i}^{t+1}$$ (25)

The optimizer will find the best weight vector to reduce the validation loss through the use of iterative updates.

The best modality weights are received as shown in Equation 26:

$$a^{*}=\arg\min_{a}L(a)$$ (26)

Where *L(α)* is the loss of classification based on the fused representation.

#### 3.4.2 Optimization-driven multimodal feature fusion

After having the best weight vector *α^∗^* the best weighted mix of the modality features is obtained in Equation 27:

$$h_{\text{fusion}}=\sum_{\mathrm{m\epsilon M}}a_{m}^{*}h_{m}$$ (27)

Where *h_fusion_* is the fused multimodal representation which has complementary information of text, audio and video modalities.

Such an optimal fusion makes sure that modalities with a stronger discriminative information have a greater contribution to the final representation.

#### 3.4.3 Classification using fully connected layers

A two-layer fully connected network is used to classify sarcasm, and the fused multimodal representation is given to the network. To learn the nonlinear decision boundaries, a nonlinear activation function is added between the two fully connected layers in the fused multimodal representation. The first hidden transformation is given by Equation 28:

$$z_{1}=W_{1}h_{\text{fusion}}+b_{1}$$ (28)

Where *W_1_* and *b_1_* are learnable parameters.

A ReLU activation is subsequently applied to make the classifier in Equation 29 nonlinear:

$$h_{1}=\text{ReLU}(z_{1})$$ (29)

Where *ReLU(x) = max(0, x).*

The activated hidden representation is subsequently projected into the sarcasm label space by the second fully connected layer as shown in Equation 30:

$$z_{2}=W_{2}h_{1}+b_{2}$$ (30)

Where *W_2_* and *b_2_* denote the learnable parameters of the output layer.

Finally, the output of the logits is passed through the Softmax activation function to obtain the probability distribution over the sarcasm classes as in Equation 31:

$$p_{\text{final}}=\text{softmax}(z_{2})$$ (31)

Where *p_final_ ∈ R^C^* and *C = 2* represents the two categories of sarcasm (sarcastic and non-sarcastic).

The nonlinear activation allows the classifier to learn the complex interaction between the fused multimodal features, yielding better representation ability than using one linear transformation.

#### 3.4.4 Objective function for model training

The general model is trained on the categorical cross-entropy loss between the ground-truth sarcasm labels and the predicted probabilities as shown in Equation 32:

$$L=-\sum_{c=1}^{C}y_{c}\log(p_{\text{final},c})$$ (32)

Where *y_c_* represents the one-hot encoded ground truth label.

At the optimization phase, PSO does not use the raw high-dimensional inputs but works on the feature representation of the trained unimodal models. This greatly decreases the computational complexity but allows optimal modality weight search to be successful. Thirty candidate particles was the number of swarm population and the maximum iterations per optimization cycle was to be 50. The candidate weight vectors were all required to be non-negative and sum to one.

The convergence was normally reached in 30–40 iterations and then the best weight vector was utilized to obtain the final multimodal representation. PSO uses only compact feature embeddings as opposed to full neural network parameters, resulting in a small computational burden. In the inference step, the optimized weights of the modality are directly used to calculate the fused representation and then do classification, which can effectively predict sarcasm, and can be applied effectively to conversational AI in real-time and human-computer interaction systems.

#### 3.4.5 Optimization workflow and computational complexity

There are two successive stages in the optimization process. During the first stage, the modality-specific encoders RoBERTa, HuBERT and ViViT are jointly trained on the training set, without any objective but to learn a representation for the textual, acoustic and visual features. However, once convergence, the number of encoder parameters will be fixed and does not change in the subsequent optimization. Consequently, the representation of the modality learned becomes no longer updated and the pretrained transformer models are not updated during the optimization of PSO.

In the second stage, only the optimal modality weight vector *(α_text_, α_audio_, α_video_)* is solved by Particle Swarm Optimization (PSO). Every particle is a single possible configuration of the weights that would meet the constraint, *α_text_ + α_audio_ + α_video_ = 1*. The modality embeddings obtained from the frozen encoders are fused together using a weighted fusion strategy as defined in Equation 27 for each candidate particle. The fused representation is then passed through the fully connected classifier, having fed the corresponding parameters into the training set; the modality weights are kept as fixed for that particular particle. This categorical cross entropy loss is then assessed on the validation set and becomes the fitness value for the particle. As a consequence, the use of the validation set is solely for finding the weights of the candidate modalities, while the test set is completely unseen until the end of the process, thus separating optimization and evaluation.

In each step, the velocity and location of the particle are changed using Equations 24, 25. Each update the modality weights are normalized to meet the simplex constraint and the fused representation is recalculated. If the validation loss of the particle is lower than the previous validation loss, then the personal best of the particle is updated. Likewise, whenever one particle finds a better validation loss than currently best global solution, the global best particle is updated. This optimization process takes place again and again until max. Number of iterations is obtained or global best solution is converged.

Conventional end-to-end optimization will not update millions of parameters of the pretrained transformer encoders during PSO optimization, which is a major difference from the proposed framework. Rather, the three modality weights are optimized and the encoder parameters are kept fixed, significantly narrowing down the scope of search for the optimization. The computational burden of PSO is only added by repeated evaluation of the fused feature representations and the associated classifier loss after the training of the encoder. For our implementation, the number of particles in the swarm was kept at 30, with number of iterations set to 50, and convergence reached around 30–40 iterations.

The optimization problem is a simple 3-dimensionalization problem; however, the objective function is a nonlinear optimization landscape because of the interaction among the multimodal feature representations and the downstream classifier. The proposed PSO-based optimization was chosen because it is a derivative-free global search method which can be easily extended to higher dimensional multimodal fusion problems without changes to the optimization framework. However, another line of investigation would be to compare with other optimisation methods like grid search or random search.

#### 3.4.6 Distinctive features of the proposed PSO-based multimodal framework

Although cross-modal attention-based approaches are proven to be effective in recent multimodal sarcasm detection (MSD) methods, most of these methods are tightly coupled to the network architecture and the attention weights are fixed throughout the end-to-end training. This kind of fusion is mainly based on pairwise modalities interactions, and has no explicit optimization of the overall contribution of every modality in different conversational scenarios. The problem of multimodal fusion, however, is considered as an optimization problem here and PSO is applied to learn the optimal weights for the visual, acoustic and textual representations generated by the models RoBERTa, HuBERT & ViViT, respectively. The proposed strategy is a PSO-based search method that looks for the best-found fusion solution with minimum classification loss to make the strategy robust in case of noisy, ambiguous or incomplete information in the modalities.

The modality-weight optimization was performed using a population-based search procedure to ascertain the relative contributions of the visual, acoustic, and textual representations. A candidate solution was represented by a three-dimensional weight vector *α = [α_text_, α_audio_, α_video_]*, corresponding to the text, audio, and video modalities, respectively. At each optimization iteration, the candidate weights were randomly initialized as non-negative values and normalized such that *α_text_ + α_audio_ + α_video_ = 1*, thereby ensuring that the resulting weights remained within the feasible search space. For each candidate weight vector, a fused representation was generated according to *F = α_text_F_text_ + α_audio_F_audio_ + α_video_F_video_*, where *F_text_, F_audio_, F_video_* and denote the modality-specific feature representations extracted by RoBERTa, HuBERT, and ViViT, respectively. The quality of each candidate solution was evaluated using the F1-score obtained from the corresponding fused representation on the validation set, and the candidate producing the highest validation F1-score was retained as the best-found modality-weight vector. The optimization was performed for a maximum of 50 iterations, to ensure reproducibility with a fixed random seed of 42. The non-negativity and unit-sum constraints were reapplied to every candidate weight vector to prevent invalid modality contributions. The optimization was terminated after the predefined 50 iterations, after which the best-found modality weights were fixed and used to construct the fused representation for the subsequent classification and inference stages. No modality-weight updates were performed on individual samples or during inference. Since the optimization operated only over three modality weights rather than the parameters of the pretrained encoders, the search remained compact and did not require modification of the internal architectures of RoBERTa, HuBERT, or ViViT.

Proposed framework is fusion at the feature level, but not at the decision level. To this end, the modality-specific feature representations learned by RoBERTa, HuBERT and ViViT are first projected into a shared feature space, and then fused together by the optimized modality weights to obtain a unified feature representation. The final prediction of sarcasm is then passed by this fused representation to the fully connected classification layers. Decision-level fusion, on the other hand, merges the independent predictions or class probabilities made by the independent modality-specific classifiers, such as by voting, averaging or weighted combination of decisions. The proposed framework allows the PSO-based optimization to work directly on the modality-specific feature representations, prior to the final classification stage, where the final decisions of each modality are not fused. Thus, the proposed approach is a feature-level multimodal fusion approach with optimization-based modality weighting.

Graph-based multimodal sarcasm detection models typically build multimodal or heterogeneous graphs, which focus on intra-modal and inter-modal relations between visual, acoustic and textual features. These approaches are effective for modeling structural relationships but can be complicated to implement, involve graph attention mechanisms and entail heavy computational costs, especially in the case of large conversational datasets. Moreover, graph representation is very sensitive to graph topology design and message-passing strategies, so that graph constructions affect the performance of the graph representation. The proposed framework overcomes the above limitations by directly optimizing complementary modality contributions via a compact PSO-based fusion mechanism on high-level transformer embeddings. The architecture is optimized and achieves a fusion without the need for explicit graph modeling while maintaining discriminative information from all modalities and, as a result, is low computational and easy-to-train, easy-to-generalize, and easy-to-deploy for practical multimodal application of sarcasm detection.

Recently, LLMs and MLLMs have displayed outstanding performance in sarcasm detection by large-scale pretraining and instruction tuning. These models, however, are typically expensive to infer, demand extensive parameters, and are difficult to run in real-time human–computer interaction systems or resource-limited settings. Moreover, the majority of LLM-based methods use implicit reasoning and do not optimize the relative importance of feature modalities during feature fusion. The novelty of the proposed work is not the novelty of using new transformer encoders, but of combining the ones that are already well established for text, audio, and video with an optimization-based fusion network which explicitly determines the optimal contribution of the video, audio, and text features. This adaptive PSO-based multimodal fusion is an efficient, interpretable, and computation-economical approach with effective sarcasm detection results, which is well suited for a practical optimization between prediction accuracy, robustness, and deployment efficiency.

It is essential to note that the main value of the proposed framework is not the creation of a new Particle Swarm Optimization algorithm, but the formulation of the multimodal transformer fusion as an explicit optimization problem in the context of sarcasm detection. In comparison to traditional multimodal fusion approaches which use fixed fusion rules or concatenation or attention mechanisms whose modality contribution is implicitly learnt in the network during training, the proposed approach decouples feature extraction from modality-weight optimization. High-level contextual embeddings are produced by RoBERTa, HuBERT and ViViT, which are first trained independently and then aggregated by an explicit constrained optimization over all embeddings, holding the validation classification loss to be minimized. The decoupled optimization approach allows adaptation of the relative importance of the textual, acoustic, and visual modalities without changing pretrained transformer architectures and adding any extra cross-modal network complexity. The proposed methodology therefore offers an optimization-guided multimodal fusion formulation that is easily interpretable, easy to be extended into other multimodal classification problems, lightweight in terms of computation, and has maintained a good performance in sarcasm detection.

The novelty of the proposed framework is not in the fully replacing of the existing pretrained encoders, but in proposing an optimization based multimodal fusion mechanism, independent of the feature extraction backbone. RoBERTa, HuBERT and ViViT represent textual, acoustic and visual information, respectively, in a very discriminative way, and usually they are fused by means of fixed weighting or learned attention mechanisms during training the network models. Let us consider in contrast the proposed framework which uses an explicit optimization procedure, Particle Swarm Optimization, to find the best-found modality weights for the classification loss. This can separate the feature extraction process from the fusion process, and the feature fusion part is not optimized, thus keeping the strong modality-specific representation and adaptively choosing the relative importance of features for sarcasm detection. This approach can therefore be interpreted more clearly, is computationally more efficient, and is more robust than the original transformer architecture, while maintaining the simplicity of the transformers themselves.

## 4 Data description

The current research applies the Multimodal Sarcasm Detection Dataset (MUStARD), a benchmark dataset that is specially created with multimodal sarcasm detection tasks. The data is synchronized with both video and audio as well as textual transcripts, which makes it very appropriate in the study of sarcastic expressions as a multimodal learning system. Sarcasm can be subtly interacting between vocal tone and linguistic content, as well as between facial expression and linguistic content, hence a dataset that combines all these modalities is necessary to create effective sarcasm detection mechanisms. MUStARD offers annotated conversation clips taken out of sitcoms on the television where each speech is annotated as being sarcastic or non-sarcastic, which allows supervised learning of sarcasm classification.

The MUStARD dataset is based on conversational extracts of television shows that are popular like Friends, The Big Bang Theory, and The Golden Girls, in which sarcastic expressions are common in everyday speech. Every video clip is associated with a particular utterance made by one of the characters in a dialog. The data comprises a set of synchronized.mp4 video files, audio cues, and text transcripts of each utterance. Such multimodal samples can enable models to simultaneously include linguistic information in text, prosodic information in speech and visual information including facial expressions and gestures, which can be significant in detecting sarcastic intent. In contrast to a lot of text-only sarcasm datasets, MUStARD extends audiovisual information explicitly, thereby making it possible to study sarcasm in a realistic conversation context.

The sample size will include about 690 multimodal utterances, with each clip being a conversational chunk labelled as sarcasm. The annotations were filtered by hand to achieve reliability and sample-to-sample consistency. Every sample has contextual dialog before the target utterance, as well, which is critical to detection of sarcasm because sarcastic meaning is commonly contingent on the conversational context. This contextual information allows models to learn contradiction between literal utterance and intended meaning which are some of the frequent signs of sarcasm.

Besides multimodal content, MUStARD offers speaker information and dialog context, which can be used to capture pragmatic features of sarcastic communication. The dataset allows thus broad modeling of sarcasm using textual semantics, acoustic prosody, and visual facial dynamics. This multimodal design enables MUStARD to be used as a suitable dataset to test the efficacy of the advanced deep learning models such as HuBERT to learn audio representations, ViViT to learn spatio-temporal video representations, and RoBERTa to learn contextual text representations, all of which are incorporated into the proposed multimodal sarcasm detector.

Table 1 summarizes the distribution of sarcastic and non-sarcastic utterances in the MUStARD dataset in classes, which provides an overview of the dataset composition on which the proposed model was trained and evaluated.

**Table 1:** Distribution across classes in the MUStARD dataset.

Class	No. of clips	Proportion
Sarcastic	345	0.50
Non-Sarcastic	345	0.50
Total	690	1.00

The comparably equal ratio between sample classes guarantees that the model can best learn discriminative features without being biased toward a specific class. MUStARD is a popular benchmark dataset with synchronization audio, video and textual modalities as well as contextual dialog information and is highly used to test multimodal sarcasm detection systems.

## 5 Implementation details

The multimodal sarcasm dataset (MUStARD) is a standard multimodal sarcasm dataset that is used for detecting sarcasm and is synchronized in audio, video, and text modalities provided in the current paper. The data is represented by each sample, which is a conversational utterance in the sitcom shows and it is labelled as either sarcastic or non-sarcastic. The multimodal analysis of sarcasm is done comprehensively because the data set includes consistent video clips, speech signals and text transcripts. Before training the model, all modalities were pre-processed using the standardized procedures described in the previous sections, which ensured that all modalities had the same sampling rate, frame size and sequence length.

A graphics card with 24 GB VRAM (such as an NVIDIA RTX 4090) was selected to handle the experiments, due to its fast data storage and processing capabilities. The graphic card used for the experiments was of NVIDIA RTX 4090 (24 GB VRAM), as it allows for fast data storage and processing. All software was installed on Windows 11 with CUDA 12.3 & cuDNN acceleration and Python 3.11, PyTorch 2.3, HuggingFace Transformers, NumPy, Torchaudio, NumPy & OpenCV were used. All models were therefore trained with mixed-precision computation to maximize usage of the GPU and improve the training speed, while maintaining the numerical stability. The extracted modality-specific feature representations are not optimized directly by the proposed method based on PSO fusion, which is based on the pretrained encoders. In the current research, however, the study does not quantitatively estimate the number of trainable parameters, the number of Floating-Point Operations (FLOPs), peak memory consumption on the GPU, and training time and inference time. Therefore, the lightweight, computational efficiency, real-time applicability and/or low delay of the proposed framework are not definitive conclusions. In the future, a detailed computational efficiency analysis was taken into account.

### 5.1 Experimental design and data splitting methodology

A speaker-independent experimental protocol was implemented to assess it fairly, reproducibly, and leakage-free. Voice tone, speaking style and facial appearance are some of the characteristics of the speaker that are very sensitive to sarcasm detection models. Thus, when comparing training and testing sets it is necessary to avoid speaker overlap so that the performance can be inflated because of memorization of the speaker-specific patterns.

#### 5.1.1 MUStARD Split strategy

The MUStARD dataset comprises of 690 multimodal conversational utterances from a number of television sitcoms that have been synchronized with textual transcripts, speech signals and video clips. The characters utter a number of sentences and the data has been split at the utterance level for training, validation and testing.

The whole dataset was split into 3 sections: training set (60%), development set (20%) and test set (20%) based on the volume of the utterances. The total number of utterances in the training set is 414 utterances (60%), 138 utterances (20%) in the validation set and 138 utterances (20%) in the test set, which is a total of 690 utterances (100%). The training set only was used for training the model and for hyperparameter optimization, the validation set was used, and the test set was used only for the final evaluation of the model. The confusion matrix is therefore a 138 × 138 matrix (for the 138 utterances in the held-out test set), rather than a 690 × 690 matrix (for the 690 utterances in the test set).

In the partitioning stratified sampling was used to guarantee that the distribution of sarcastic and non-sarcastic utterances in the 3 subsets was roughly equal and that the class distributions in the 3 subsets were roughly similar. The weights tuning and optimization in the fusion process were validated using a validation set and only a test set was used to evaluate the final performance of the hyperparameters of PSO algorithm.

Conversational clips were taken from several television sitcoms and are included in the MUStARD dataset, where they vary in style of dialog, visuals, speaker characteristics, and recording conditions. Since the dataset was partitioned at the utterance level rather than at the speaker or dialog level, speaker identities were not explicitly separated across the training, validation, and test sets. Therefore, the present evaluation does not guarantee complete speaker-independent or dialog-independent generalization, and potential speaker or dialog overlap across the partitions cannot be completely excluded. However, the test set remained fully held out during model training and PSO-based hyperparameter optimization. Speaker- and dialog-independent partitioning and its effect on generalization are important directions for future investigation.

#### 5.1.2 Training methodology and performance assessment

The training split is used to train each modality-specific encoder (HuBERT, ViViT and RoBERTa). Hyperparameter tuning and determination of the best multimodal fusion weights were done on the validation split by the Particle Swarm Optimization (PSO) algorithm. No further tuning of the parameters has been done and the last model has only been tested once on the test set.

In order to guarantee statistical stability, each experiment was run with several random seeds (three runs). The values of the evaluation metrics are then shown as mean and standard deviation of these runs. This step minimizes the impact of haphazardness due to weight initiation and stochasticity of training.

#### 5.1.3 Reproducibility measures

In order to be able to reproduce the results of the experiment, a number of precautions were observed:

- Yet, predefined speaker lists were used to determine data splits.
- All preprocessing parameters (sampling rate, sequence length, frame resolution) remained unchanged in all experiments.
- Every model was trained with the identical optimizer (AdamW), the learning rate scheduler (linear warm-up, followed by cosine decay), the dropout rate (0.3), and the weight decay (1×10^−5^).
- The use of macro-averaged recall, precision & F1-score to report performance metrics was to address the possibility of class imbalance.

The exact pretrained checkpoints and fine-tuning configurations used in the experiments were kept consistent across all runs. The audio modality used HuBERT-Base (facebook/hubert-base-ls960), pretrained on the LibriSpeech dataset, with AdamW, a learning rate of 1 × 10^−4^, dropout rate of 0.3, weight decay of 1 × 10^−5^, and a maximum of 30 epochs with early stopping (patience = 5), using a linear warm-up followed by cosine decay learning-rate scheduling. The text modality used RoBERTa-Base (FacebookAI/roberta-base) with AdamW, a learning rate of 2 × 10^−5^, batch size of 32, dropout rate of 0.3, weight decay of 1 × 10^−5^, and a maximum of 30 epochs with early stopping and the same linear warm-up followed by cosine decay schedule. The video modality used ViViT-Base (google/vivit-b-16×2-kinetics400) with AdamW, a learning rate of 1 × 10^−4^, batch size of 16, dropout rate of 0.3, weight decay of 1 × 10^−5^, and a maximum of 30 epochs with early stopping and cosine decay scheduling.

### 5.2 Model architecture and configuration settings

The audio channel used the HuBERT-Base model, which was trained on the LibriSpeech dataset. It is a model that maps raw audio waveform to high level acoustic representations by passing them through a convolutional feature encoder and a contextual encoder derived from Transformer. The prosodic elements such as pitch, tone and rhythm that can be embedded, for example, to identify sarcastic speech. The contextualization of the frame-level embeddings was followed by a fixed sized representation, which was sent to a classification layer. AdamW, a learning rate of 1 × 10^−4^, dropout rate of 0.3, and weight decay of 1×10^−5^ were used to optimize the audio encoder. Early stopping (patience = 5) and linear warmup were used during training and cosine decay learning rate schedule (30 epochs) were used.

For the text modality, the RoBERTa-Base model was used, as it has high contextual ability of language understanding. The tokenizer divides transcripts into subword tokens with the help of Byte Pair Encoding (BPE). The contextual embedding of the [CLS] token of the last Transformer layer was obtained as the utterance representation. This was represented in a fully connected layer to make predictions of sarcasm labels. AdamW is used for training the text encoder with learning rate = 2×10^−5^, batch size = 32, dropout rate = 0.3, weight decay = 1×10^−5^. The number of epochs trained was fixed at 30, but was early stopped per the same warm up and cosine decay as other modalities.

The video modality was based on ViViT (Video Vision Transformer) architecture that modeled spatio-temporal visual cues that included facial expressions and gestures of sarcasm. Video clips were sampled at 25 FPS, reduced to 224 × 224, and normalized to the [0,1] range, and broken down into 16-frame segments. The ViViT encoder produced spatio-temporal embeddings that were contextualized and comprised of facial dynamics and motion patterns. The extracted feature representation was fed into a classification head to make sarcasm predictions. AdamW was used to perform video training, with a learning rate of 1 × 10^−4^, 16 batch size, 0.3 dropout rate, and 1 × 10^−5^ weight decay, and 30 epochs with early stopping and cosine decay scheduling.

### 5.3 Fusion strategy

The HuBERT, RoBERTa, and ViViT encoders extracted features representations after unimodal training on the penultimate layers. These modality-specific embeddings were subsequently given to a Particle Swarm Optimization (PSO) module to decide the best modality weighting scheme.

PSO is a population-based algorithm that calls on an initial population of candidate weight vectors which are potential combinations of modality weights. All candidate solutions are assessed in terms of the classification loss of the fused representation on the validation set. In every iteration, the particles move based on the standard PSO update equations which balance exploration of search space and exploitation of promising solutions.

The optimizer uses the candidate weight vectors to generate a single optimized modality weight vector *α^∗^* for the multimodal framework at a point of convergence. These modality weights are learned globally rather than separately for each batch or individual sample. A weighted sum of the modality embeddings is then calculated to obtain the fused feature representation. It is the fused representation that is propagated with two fully connected layers, which present the last sarcasm classification module. The classifier provides the most likely probability of the two sarcasm categories (sarcastic and non-sarcastic).

PSO optimization was done with 30 particles and maximum 50 iterations per optimization cycle. All candidates weight vectors were non-negative and added up to one. The extra computational cost is not very large since PSO uses compact feature representations instead of complete neural network parameters.

Inference is performed by keeping the optimized global modality weights constant for all test samples, batches, and inference instances. The optimized weights are not updated during inference, and the resulting prediction is generated using the weighted fused representation and the fully connected classification layers. Thus, the modality weights are learned globally using the validation set during the PSO optimization stage and subsequently fixed for inference.

### 5.4 Evaluation measures for model performance

The impact of the suggested multimodal sarcasm detection model was checked by conventional classification measures such as accuracy, precision, recall, F1-score, and confusion matrix analysis.

The proportion of the correctly classified samples overall is called accuracy and is defined in Equation 33:

$$\text{Accuracy}=\frac{\mathrm{TP}+\mathrm{TN}}{\mathrm{TP}+\mathrm{TN}+\mathrm{FP}+\mathrm{FN}}$$ (33)

*TP*, *TN*, *FP*, and *FN* are the true positives, true negatives, false positives, and false negatives, respectively.

Precision is the number of samples that are correctly predicted relative to the number of samples that are predicted in a particular class, *c* as shown in Equation 34:

$$P_{c}=\frac{TP_{c}}{TP_{c}+FP_{c}}$$ (34)

Equally, recall is a measure of the proportion of correctly predicted samples of the total number of samples that is in class *c* as shown in Equation 35:

$$R_{c}=\frac{TP_{c}}{TP_{c}+FN_{c}}$$ (35)

The F1-score is the harmonic mean of precision and recall as shown in Equation 36:

$$F1_{c}=\frac{2.\;\;P_{c}.R_{c}}{P_{c}+R_{c}}$$ (36)

The end precision, recall and F1-score of the two sarcasm classes were computed using macro-averaging to make sure that both categories contribute equally to the end result.

Moreover, a confusion matrix was built to examine the performance of the prediction in classes as in Equation 37:

$$\begin{array}{l} CM_{i,j}=\text{Number of samples with true}\;\\ \text{class}\;i\;\text{predicted}\;\mathrm{as}\;\text{class}\;j \end{array}$$ (37)

The confusion matrix gives the information on systematic misclassifications of sarcastic and non-sarcastic utterances, which allows the analysis of the model behavior in the context of various conversational situations.

## 6 Results and discussions

The multimodal approach for sarcasm detection was tested on the three modalities: textual, acoustic and visual. The encoders used were RoBERTa for representing the context of textual data, HuBERT for extracting acoustic features, and ViViT for representing the spatio-temporal structure of video. The features from these pre-trained transformer models were adaptively fused with the proposed Particle Swarm Optimization (PSO)-based fusion strategy which optimally selects the features from each modality to be used for sarcasm classification. Accuracy, Precision, Recall, and Weighted F1-score were used to assess the proposed framework performance. Comprehensive assessment of the effectiveness, robustness and contribution of the proposed PSO-based multimodal fusion framework was also carried out by employing confusion matrices, training-validation accuracy curve, a comparison of the proposed method with the existing state-of-the-art methods and an ablation study of different fusion strategies.

### 6.1 HuBERT model

HuBERT model was trained to produce rich acoustic representations of the prosodic, spectral and temporal features of raw speech signals. The model shows high results in detecting sarcasm, which is evidence of how well audio-based features detect subtle tonal differences linked to sarcasm speech. The total outcome has a recall of 0.88, precision of 0.88, F1-score of 0.88, and an accuracy of 0.88, which means that the acoustic cues on their own form a strong baseline of sarcasm classification.

Further analysis of the performance in the classes in Table 2 gives more information about the model behavior. The Sarcastic class has a larger recall of 0.91 and this implies that the model is very effective at detecting sarcastic utterances and this could be attributed to the unique prosodic features including pitch variations, stress and intonation patterns. Conversely, the Non-Sarcastic group obtains a somewhat lower recall of 0.84, which implies that some non-sarcastic samples are falsely classified as sarcastic occasionally. This can happen because of the overlapping acoustic features in which neutral or expressive speech can be similar to the sarcastic tone.

**Table 2:** HuBERT classification performance on the MUStARD dataset.

Sarcasm classes/aggregate	Recall	Precision	F1-Score
Sarcastic	0.84	0.88	0.86
Non-Sarcastic	0.91	0.88	0.90
Macro Avg	0.87	0.88	0.88
Weighted Avg	0.88	0.88	0.88

The diagonal values (63 and 59) in Figure 6 show the right predictions of Non-Sarcastic and Sarcastic classes respectively, which indicates high classification. Nevertheless, 8 cases of non-sarcastic samples being classified as sarcastic and 8 cases of sarcastic samples being classified as non-sarcastic also occur. This unequal distribution is indicative of the model being slightly biased toward detecting sarcasm which correlates with the higher recall of the sarcastic class.

**Figure 6:**
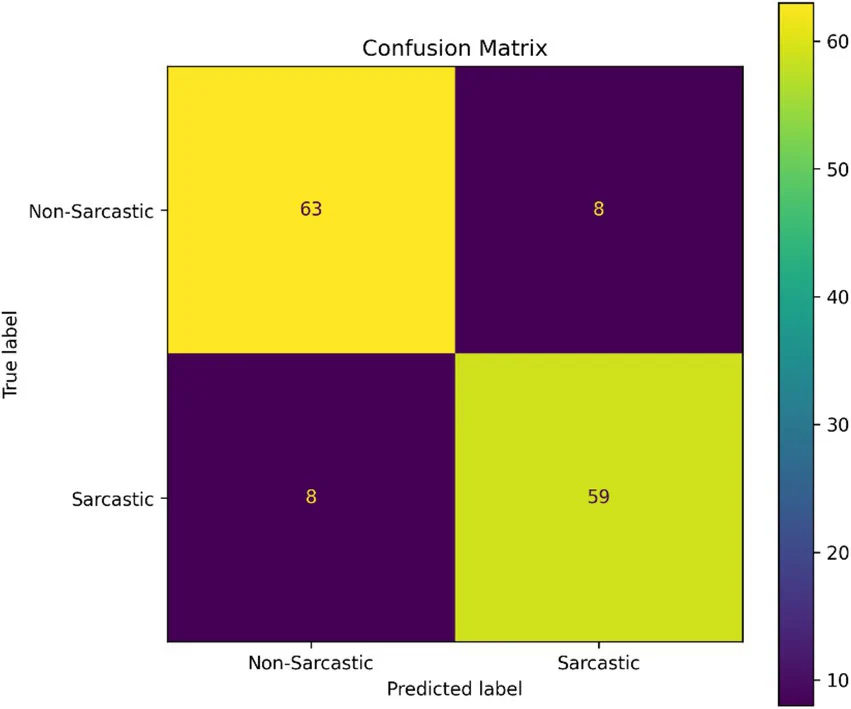
HuBERT model classification confusion matrix.

This misclassification can be explained by the ambiguity in the vocal expressions as some neutral or slightly expressive tones can sound as sarcastic speech. Equally, there are sarcastic statements that do not have firm prosodic indicators, which can be interpreted as non-sarcastic. These trends suggest that the audio features can work well to represent tonal intensity, but can fail to respond to context-specific sarcasm.

In Figures 7, a smooth and gradual convergence with epochs takes place. First, there are significant fluctuation in the validation accuracy, which is the attempt of the model to generalize to a few patterns at the beginning of training. Both curves move upwards with the course of training and start to level off, which is an indicator of successful learning of the discriminative acoustic features.

**Figure 7:**
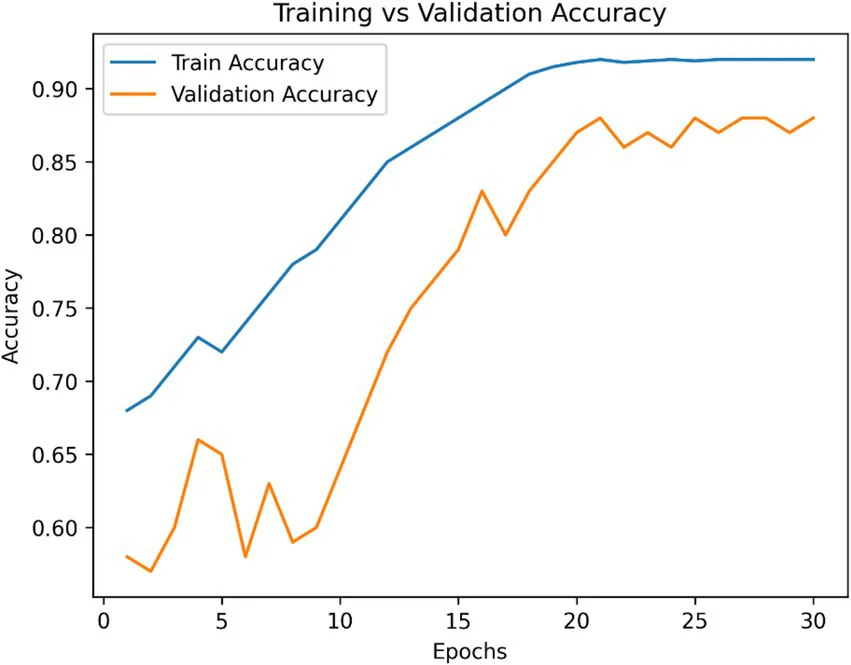
Accuracy trends during HuBERT model training.

The difference between both accuracies is low during the training process, and it indicates low overfitting, and a high level of generalization. Periodic overlaps of the curves also show realistic training dynamics in which validation performance is temporarily larger than training accuracy because of stochastic optimization and variability of data.

The last convergence at about 0.92 (training) and 0.88 (validation) shows that the model has successfully acquired meaningful representations without learning noise in the data.

In practical terms, the findings established that audio-based sarcasm detection is very efficient, especially when sarcasm is conveyed by using strong prosodic features like exaggerated tonal, pitch, or stress patterns. The sarcastic speech recall is large, which shows that the HuBERT model is suitable in a situation where it is important to detect sarcasm, e.g., conversational AI, systems that analyze sentiment, and human-computer interaction.

Nevertheless, the resulting confusion of classes indicates that acoustic aspects might not be sufficient to depict contextual or semantic subtleties of sarcasm. Subtle or situational sarcasm which is more dependent on linguistic meaning than on tone might not easily be identified using audio signals.

Thus, the results support the significance of multimodal strategies, in which textual and visual modalities can be used to supplement acoustic characteristics to eliminate ambiguities and increase the overall strength. The combination of different modalities helps to have a more comprehensive view of sarcasm by utilizing tone, language, and facial expressions.

### 6.2 RoBERTa model

RoBERTa was trained to retrieve deep contextual textual representations of semantic, syntactic and pragmatic transcripts of language with respect to the input transcripts. The model is very effective in sarcasm detection which emphasizes the significance of linguistic indicators like contrast, exaggeration, and implicit meaning to detect sarcasm. The overall performance is 0.82 recall, 0.83 precision, 0.82 F1-score and a 0.82 accuracy, which suggests that textual features are a good starting point in the classification of sarcasm.

Further analysis of classes performance in Table 3 brings more information about the model behavior. Non-Sarcastic class has a better recall of 0.87 which indicates that the model is effective in hit-and-miss statements that are literal and straightforward and have little contextual ambiguity. Conversely, the Sarcastic group has a very slightly lower recall of 0.77, meaning that some of the sarcastic examples are falsely classified as non-sarcastic. This is possibly due to the fact that sarcasm is always dependent on the implicit context, tone or external knowledge that may not be just captured by the use of textual information.

**Table 3:** RoBERTa classification performance on the MUStARD dataset.

Sarcasm classes/aggregate	Recall	Precision	F1-Score
Sarcastic	0.77	0.87	0.82
Non-sarcastic	0.87	0.77	0.82
Macro Avg	0.82	0.82	0.82
Weighted Avg	0.82	0.83	0.82

The diagonal (55 and 58) in Figure 8 represents accurate predictions of Non-Sarcastic and Sarcastic classes respectively, which show an equal level of classification. There are however 12 cases where non-sarcastic sample is incorrectly identified as sarcastic and 13 cases where sarcastic sample is incorrectly identified as non-sarcastic. This skew implies that model is likely to overlook some sarcastic expressions, resulting in reduced recall of the sarcastic class.

**Figure 8:**
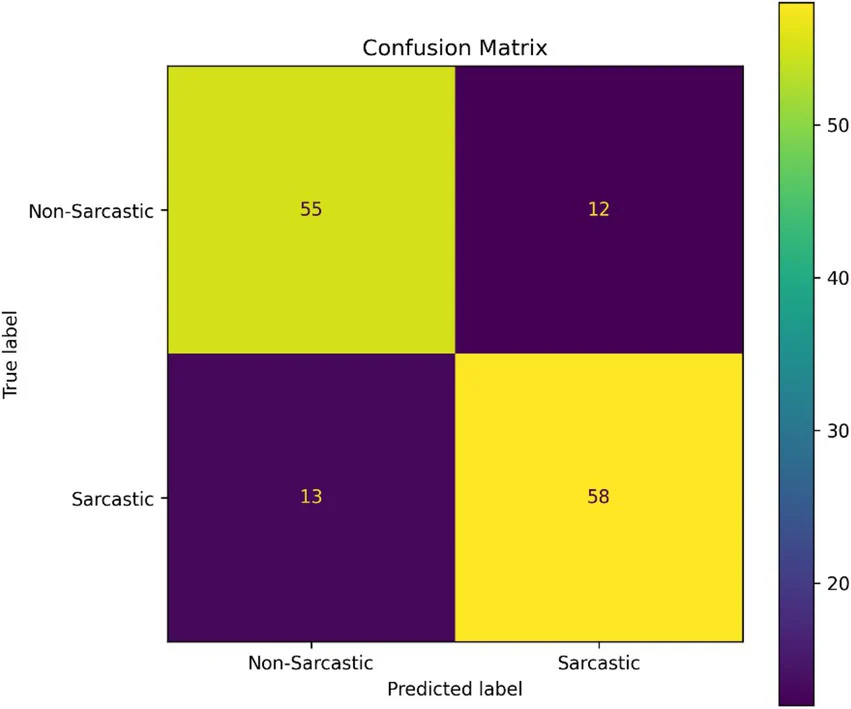
RoBERTa model classification confusion matrix.

These misclassifications can be explained by the fact that sarcasm in a text is frequently complex in nature, with meaning frequently being reliant on a context, a background, or a tone that is not directly available in the transcript. Minor sarcasm, particularly when it is made without heavy lexical indications, can seem like literal utterances. Such tendencies suggest that textual characteristics can be helpful in defining a semantic relationship, and contextual dependencies, but might be weak with implicit or pragmatically subtle sarcasm.

The curves in Figure 9 indicate a slow and fairly constant convergence to the end of the epochs. Firstly, the validation accuracy is prone to oscillation, which is an indication of the model trying to generalize to a variety of linguistic patterns and contextual variation within the dataset. The two curves exhibit a positive trend as training advances, which shows that contextual textual features are effectively learned.

**Figure 9:**
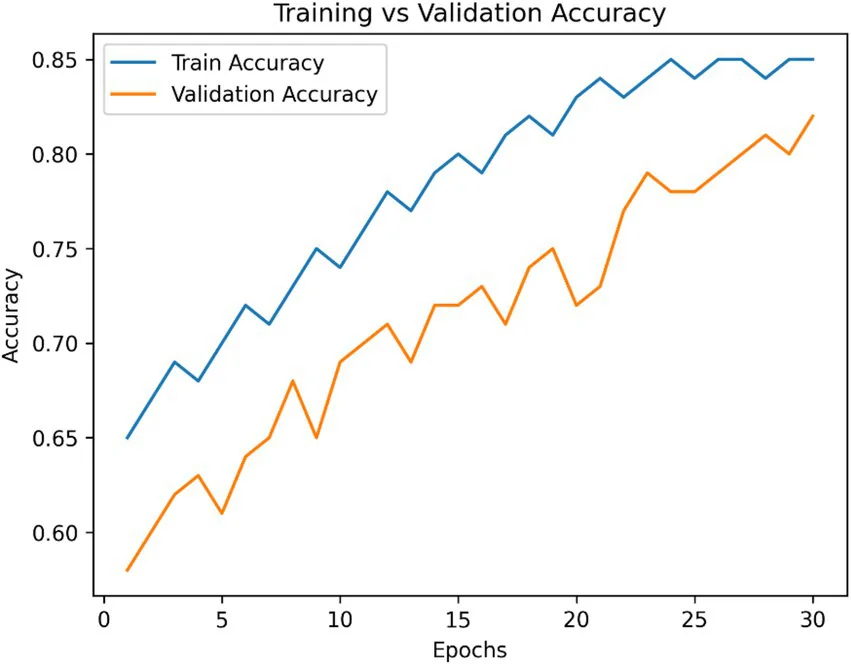
Accuracy trends during RoBERTa model training.

The difference between both accuracies is also kept within bounds, indicating that there was minimal overfitting, and there is a moderate generalization ability. Several intersections of the curves demonstrate the realistic nature of training dynamics, when the validation performance is sometimes worse than the training accuracy because of the stochastic nature of learning behavior and variance in datasets.

The last convergence to 0.85 and 0.82, means that the model is able to learn meaningful semantic representations and still generalize on unseen data.

In practical terms, the findings validate that sarcasm can be detected using textual means, and the approach is able to identify contextual and semantic cues, especially when sarcasm is explicitly represented by the means of linguistic structures, like contrast, exaggeration, or negation. The high result on non-sarcastic samples shows that model is highly applicable in detection of literal utterances in a conversation context.

Nevertheless, the comparatively lower recall of sarcastic cases indicates that text may not be so useful in capturing the whole meaning of sarcasm particularly when it is based on tone, delivery or the circumstantial context. Sarcasm which relies much on the vocal stress or even facial expression might not be well understood by text alone.

Thus, the present results highlight the significance of the multimodal integration, in which audio and visual modalities may supplement text-based characteristics to refine the performance of sarcasm detection. The combination of linguistic, acoustic, and visual information allows a deeper insight into sarcastic communication, which improves the strength of such systems in practice, e.g., conversational agents, sentiment analysis systems, and social media monitoring.

### 6.3 ViViT model

ViViT was trained on the visual representation of a video sequence, which consisted of spatio-temporal representations of facial expression, gestures, and minor motion dynamics. The model performs quite well in sarcasm detection, indicating that visual clues like facial movements, eye movement, and body language are crucial in determining sarcasm. The general performance attains a precision of 0.90, a recall of 0.90, a F1-score of 0.89 and an accuracy of 0.90, which suggests that visual features offer a very effective modality in sarcasm classification.

Further analysis of the performance by classes in Table 4 gives better insights into the behavior of the model. The Non-Sarcastic group attains a better recall of 0.96 which implies that the model is very effective in the recognition of neutral or literal visual expression in which exaggerated gestures are not present. On the other hand, Sarcastic class has a slightly low recall of 0.81 which shows that there is misclassification of some sarcastic instances as non-sarcastic. This is possibly so because some sarcastic words are mild and they might not necessarily be accompanied by an exaggerated facial or gestures.

**Table 4:** ViViT classification performance on the MUStARD dataset.

Sarcasm classes/aggregate	Recall	Precision	F1-Score
Sarcastic	0.96	0.87	0.91
Non-Sarcastic	0.81	0.94	0.87
Macro Avg	0.89	0.91	0.89
Weighted Avg	0.90	0.90	0.89

There are diagonal values of 61 and 63 in Figure 10 which show the ability to predict Non-Sarcastic and Sarcastic classes correctly, respectively. But it is 7 in both cases such that non-sarcastic samples are wrongly identified as sarcastic, and sarcastic samples are wrongly identified as non-sarcastic. Such asymmetry implies that the model is more conservative when predicting sarcasm and results in greater recall of non-sarcastic samples and lower recall of sarcastic ones.

**Figure 10:**
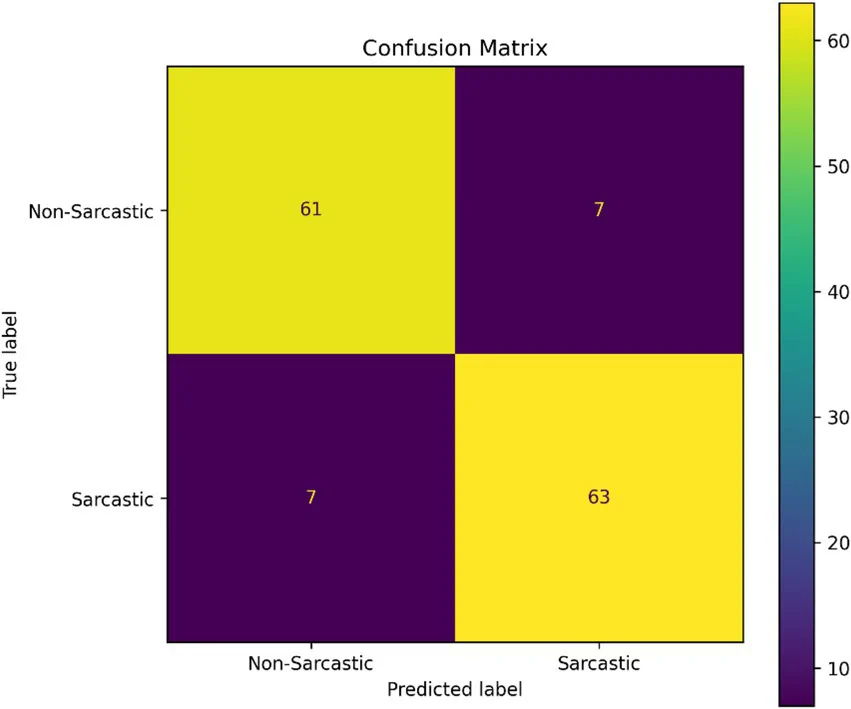
ViViT model classification confusion matrix.

These misclassifications may be explained by the fact that visual sarcasm is not always obvious, and not every sarcastic utterance is followed by exaggerated facial expressions and gestures. Sarcasm in other instances can also be expressed via tone or situation more than appearance. These trends suggest that even though the visual features prove useful in the identification of expressive and exaggerated behaviors, they might have difficulties with subtle or contextual sarcasm.

The accuracy curves in Figure 11 indicate a smooth and constant convergence during epochs. First, the accuracy of validation is not constant, it represents the diversity of visual patterns and the difficulty of acquiring spatio-temporal features. Both curves show a steady upward trend as the training continues, and it means that significant visual representations are learned.

**Figure 11:**
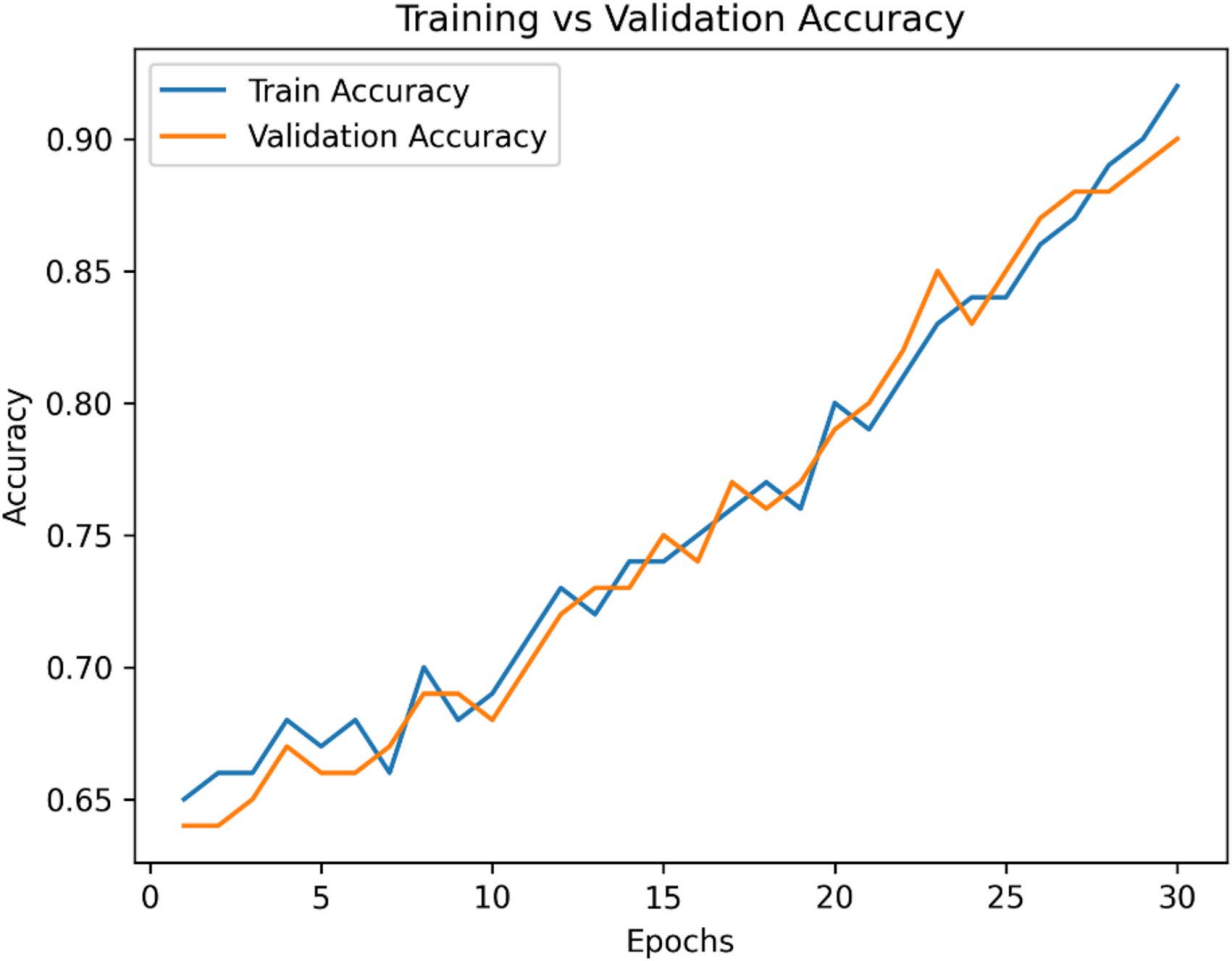
Accuracy trends during ViViT model training.

The difference between both accuracies is also low indicating low overfitting and high generalization ability. Intersections between the curves can occasionally indicate realistic training dynamics, in which the performance of validation may temporarily be higher than that of training as stochastic optimization and data variation.

This last convergence at approximately 0.90 (training) and 0.90 (validation) shows that the model learned the discriminative visual features and is also stable with regards to unknown data.

In practical terms, these findings are valid that sarcasm detection using video is very effective especially when sarcasm is conveyed using visible clues like facial expression, eye movement, or body gesture. The good recall on non-sarcastic samples says that the model is effective in recognizing both non sarcastic or literal expressions, and thus it can be used in real-world scenario involving visual analysis.

The comparatively poorer recall of sarcastic cases though does indicate that visual modality is not necessarily sufficient to represent all types of sarcasm, and that sarcasm can be expressed in subtle ways that do not have strong visual cues. When this happens, the use of visual features alone can result in omissions.

So, the findings continue to highlight the significance of multimodal integration where audio and text modalities may be used to supplement visual information to enhance the overall performance. Multimodal systems can gain a more accurate view of sarcasm by combining the expressions with tone and language context, which results in greater strength in applications like human-computer interaction, affective computing, and social media analysis.

### 6.4 PSO-based multimodal fusion with fully connected layers

The last multimodal model combines the audio, text and video feature representations with Particle Swarm Optimization (PSO) to find ideal feature weightings and then a two-layer fully connected neural network will classify. This hybrid strategy will allow the model to take advantage of the complementary information of all modalities and use acoustic, semantic, and visual information in parallel. The integrated model shows a better performance in sarcasm detection as compared to individual results on modality. The overall performance has a precision of 0.94, recall of 0.94, F1-score of 0.94 and an accuracy of 0.94, which is the effectiveness of multimodal fusion in strengthening the classification.

On a more detailed look at the performance by classes in Table 5, there are balanced and high-performance results in both classes. The Non-Sarcastic model recalls 0.96, which means that the model is very useful in the correct identification of literal statements with a low misclassification rate. Equally, the Sarcastic category has a high recall rate of 0.93 and this shows that the model effectively captures sarcasm through the combination of contextual, tonal, and visual information. The equal accuracy and recall rates between the two classes indicate the usefulness of PSO in allocating the best importance to each modality.

**Table 5:** PSO-based multimodal fusion classification performance on the MUStARD dataset.

Sarcasm classes/aggregate	Recall	Precision	F1-Score
Sarcastic	0.96	0.92	0.94
Non-sarcastic	0.93	0.96	0.94
Macro Avg	0.945	0.94	0.94
Weighted Avg	0.945	0.94	0.94

The diagonal values (67 and 63) in Figure 12 show a proper prediction of Non-Sarcastic and Sarcastic classes respectively, which shows a good classification result. The misclassification rates of non-sarcastic samples being falsely recognized as sarcastic and sarcastic samples being falsely recognized as non-sarcastic are 4 and 4, respectively, which is very low when compared to single modalities.

**Figure 12:**
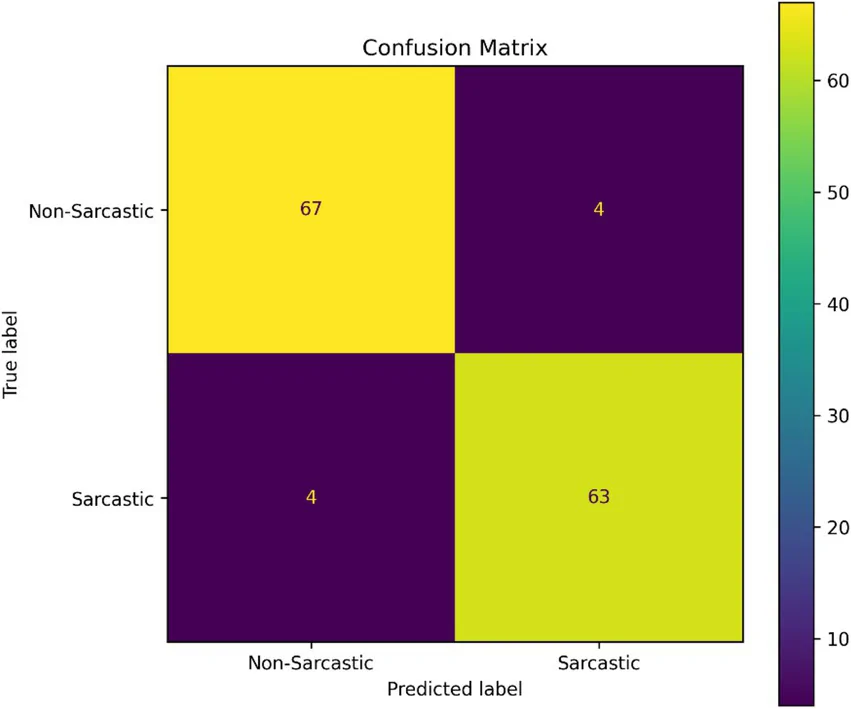
PSO-based fusion model classification confusion matrix.

This equal error distribution would indicate that the model is able to decrease ambiguity between classes because all modalities provide complementary information. The misclassifications previously witnessed in the unimodal models (i.e., confusion because of the absence of context (text), low-range tone (audio), or minimum facial expression (video)) are greatly eliminated in the fusion approach.

Practically, these findings validate that multimodal fusion can play a major role in refining the performance of sarcasm detection. The model takes advantage of the weaknesses of the separate modalities and the combination of audio, text & visual features to obtain a more detailed insight into the issue of sarcastic communication.

The accuracy and recall of the two classes are also high, which means that the model can detect sarcasm accurately and is also very strong in distinguishing between non-sarcastic content. This improvement requires the use of PSO to weight the features, which is a dynamic process in which the relative importance of each modality can be dynamically determined based on their contribution to the classification task.

Moreover, the merging with a two-layered fully connected network allows to learn the intricate relations between the multimodal features effectively and thus achieve improved decision boundaries and less classification errors.

These results emphasize that sarcasm is a multimodal phenomenon in that the meaning is produced by a combination of tone, language, and visual expression. Thus, multidimensional systems based on the use of one modality might not be able to resolve the whole intention, whereas multimodal systems offer a more consistent and scaled resolution.

Such a multimodal architecture can play a crucial role in identifying subtle human behaviors in real-world applications in conversational AI, sentiment analysis, social media monitoring & HCI, where a more context-sensitive and intelligent system is needed.

### 6.5 Impact of context in sarcasm detection

#### 6.5.1 Audio modality

Effects of contextual information on the audio modality were measured by comparing model performance in the presence of contextual inputs and in the absence of the same. These findings show that there is a great discrepancy in performance with the model having an accuracy of 88% with context and just 60% without context. This high decline brings out the significance of contextual clues in decoding prosodic characteristics like tone, pitch, and stress patterns.

With context, the model is able to reflect sarcastic intent in changes in the vocal delivery. Nevertheless, in a vacuum, a lot of sarcastic statements are aural clones of non-sarcastic speech, which contributes to more cases of misclassification. This proves that purely acoustic features cannot be used to effectively detect sarcasm without any contextual support.

The contextual confusion matrix in Figure 13 reveals a good degree of dominance in the diagonal, which indicates that both sarcastic and non-sarcastic samples are accurately classified with a few errors made. The model can differentiate tonal variation better when there is contextual information leading to balanced precision and recall of both classes. This is indicative of the fact that the model can use contextual information to disambiguate any subtle prosodic patterns.

**Figure 13:**
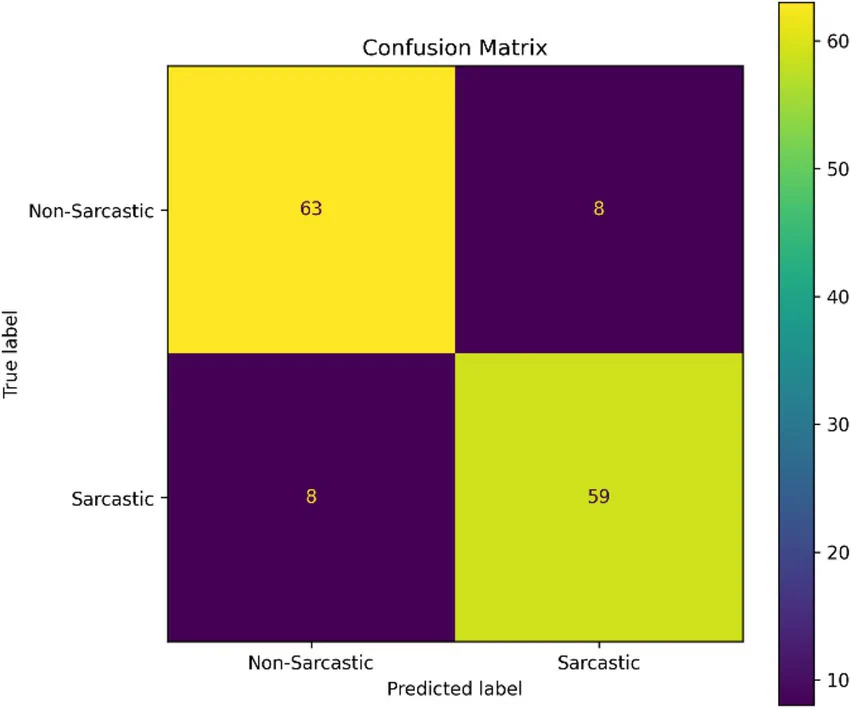
Audio modality confusion matrix (with context).

Conversely, the confusion matrix with no context in Figure 14 indicates that there are more off-diagonal values, where, more samples of sarcastic are classified as non-sarcastic, indicating that the contextual meaning is lost. The model fails to distinguish similar acoustic patterns especially when the expression of sarcasm is not exaggerated by means of tone. This shows that prosodic cues are not always discriminative enough particularly where subtle or contextual sarcasm is involved.

**Figure 14:**
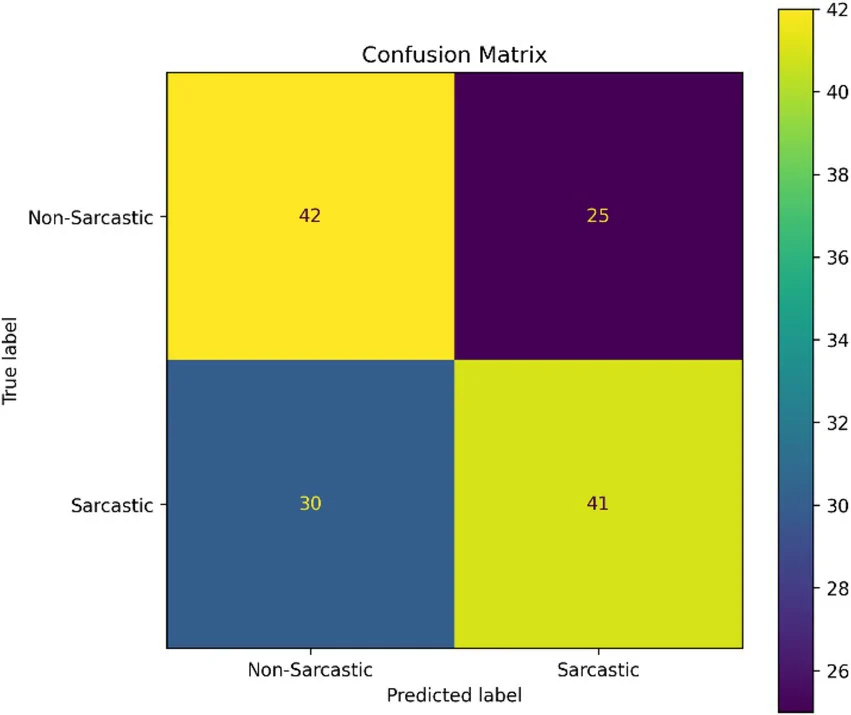
Confusion matrix for the audio modality (without context).

#### 6.5.2 Text modality

For text, the model gives an accuracy of 82% when there is context but the accuracy reduces to 55% when there is no context. This is the most significant drop in the performance of all modalities, which proves that sarcasm detection using text is extremely context-dependent.

Text sarcasm tends to be based on opposition, the preceding sentences or a hidden meaning that cannot be determined by single sentences. Sarcastic expressions can be interpreted literally without any context, hence the rate of misclassification is high. This brings out the weakness in using textual characteristics without considering the context of conversation.

The context-based confusion matrix in Figure 15 shows that both classes have balanced classification and fairly high levels of true positives that represent successful semantic comprehension. The model is effective in capturing both contextual relationships and linguistic hints like contrast and exaggeration and lets one be able to differentiate between the sarcastic and non-sarcastic cases.

**Figure 15:**
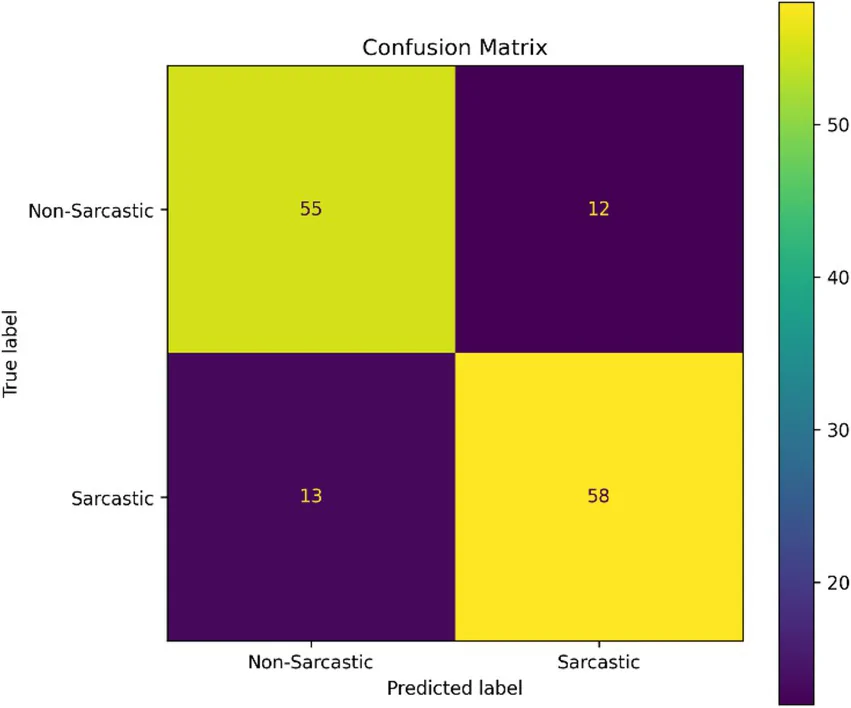
Confusion matrix for the text modality (with context).

In the absence of context, the confusion matrix in Figure 16 reveals that there is a great deal of misclassification, especially when sarcastic samples are mistakenly classified as non-sarcastic, because there are no contextual cues. This implies that a lot of sarcastic utterances lose their original meaning when taken out of context and make them understand it literally by the model. This heightened perplexity explains why discourse-level information is essential in textual sarcasm recognition.

**Figure 16:**
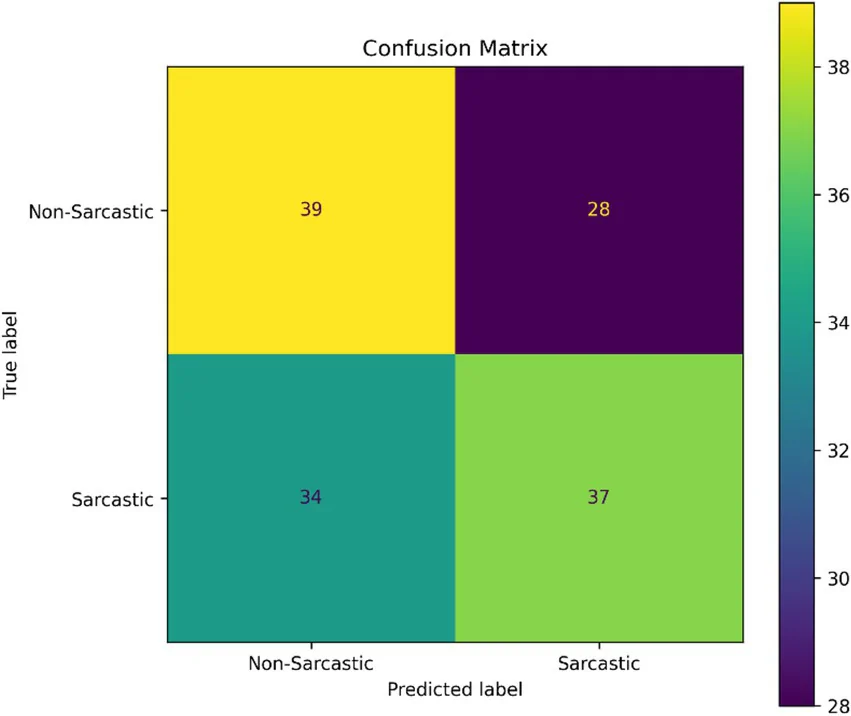
Classification confusion matrix for context-free text modality.

#### 6.5.3 Video modality

The video mode has the best performance of unimodal approaches with an accuracy of 90% with context and a lower value of 67% without context. This implies that though the use of visual clues like facial expressions and gestures are very informative, they cannot be understood accurately without the contextual information.

The model is capable of linking facial expressions and gestures with the hidden meaning of the speaker. Nevertheless, when used in isolation, subtle or neutral expressions might not necessarily point at sarcasm, resulting in imprecision and misidentification. This indicates that visual features are also powerful but not wholly self-sufficient in the detection of sarcasm.

The confusion matrix with context in Figure 17 demonstrates high accuracy and great diagonal values indicating successful recognition of sarcastic and non-sarcastic cases. The model is also advantageous in that it combines facial dynamics with contextual cues, which allow it to perform the interpretation of expressive behaviors related to sarcasm correctly.

**Figure 17:**
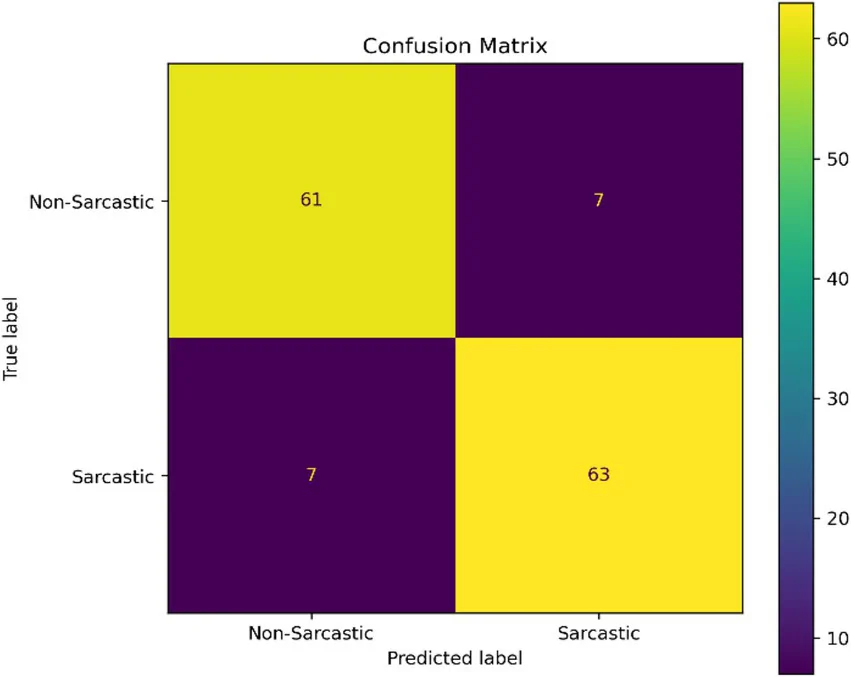
Confusion matrix for the video modality (with context).

The confusion matrix in the absence of context in Figure 18 shows more confusion among the classes, especially in sarcastic samples, which shows that it has less discriminating ability when not given contextual information. Minor facial expressions that are dependent on the context of the situation are hard to decode causing misclassification. This brings out the point that visual sarcasm tends to rely on the surrounding story and not isolated phrases.

**Figure 18:**
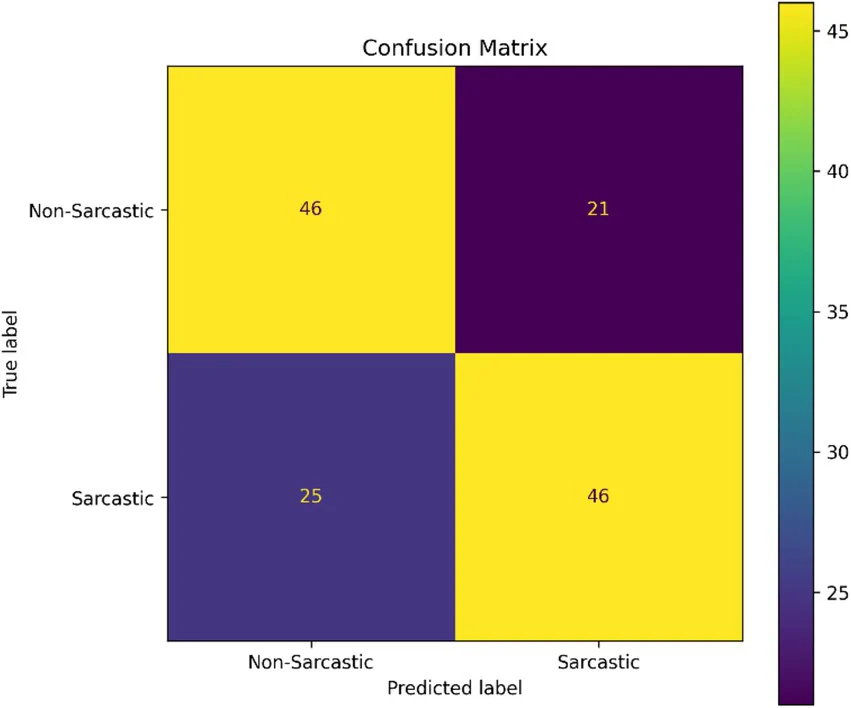
Confusion matrix for the video modality (without context).

The findings of all three modalities clearly show that context is a very critical aspect of sarcasm detection. Although both modalities record distinct elements of sarcastic communication such as acoustic tone (audio), semantic meaning (text), and facial expressions (video), none of them can record the best performance without context information.

The context modality gives the biggest decrease, which shows the significance of contextual semantics, and the decrease in audio and video modality shows that the prosodic and visual data frequently need to be interpreted in the context. These results support the fact that sarcasm is contextual and multimodal.

Altogether, this discussion supports the necessity of context-sensitive and multimodal methods and approaches, in which the integration of multiple modalities and contextual data result in more correct and reliable sarcasm detection systems that can be used in the real world.

## 7 Ablation study

To assess the effect of every modality and a multimodal combination of modalities in the suggested multimodal sarcasm detection model, extensive ablation research has been conducted. The analysis will attempt to find out the effects of the Video (V), Text (T) and Audio (A)Modalities individually and collectively. The paper proposes a training with the constant experimental condition of unimodal, bimodal and fully multimodal setups, demonstrating that the 3 modalities are complementary, and demonstrates the effectiveness of the PSO-based fusion strategy approach.

The findings in Figure 19 explicitly show that multimodal fusion is very much better than any unimodal and any bimodal configuration with the highest accuracy of 94. The unimodal methods, specifically the video modality (90%), audio (88%), and text (82%) are the most effective ones, and it is crucial to note that visual and acoustic stimuli are crucial in sarcasm detection.

**Figure 19:**
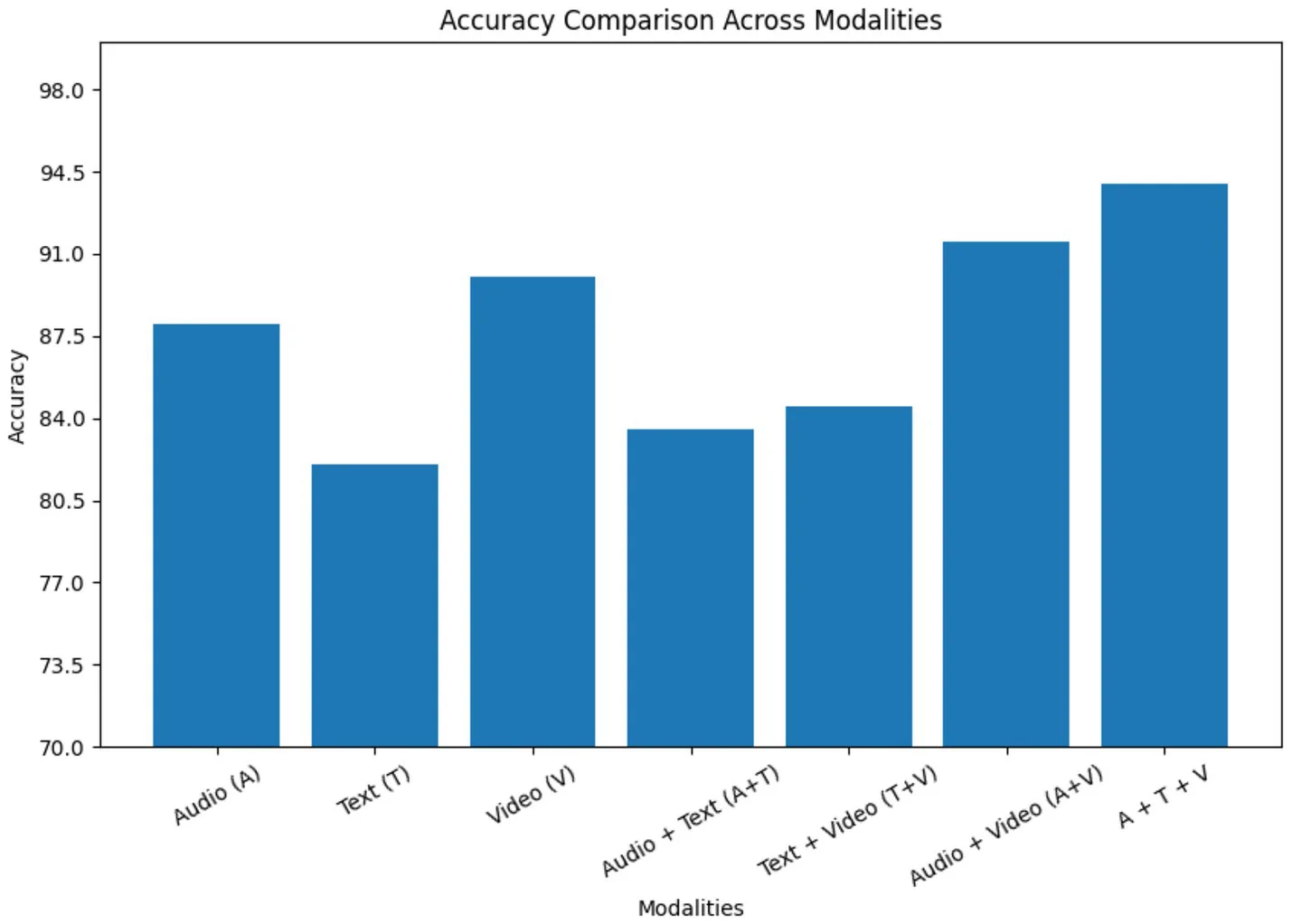
Accuracy comparison across different modality combinations.

Within the bimodal combinations, the best performance is observed with the Audio + Video (91.5%), which shows that there is a great interaction between prosodic speech characteristics and facial expressions. Contrarily, Audio + Text (83.5%) and Text + Video (84.5%) demonstrate lower performance, which implies that although the textual information is helpful in giving the contextual understanding, it does not work well when used without substantial complementary variables of audio and video at the same time.

The training curves of the various combinations of modality in Figure 20 have a steady upward trend, which suggests that learning is effective in all combinations. Nonetheless, the differences in convergence behavior indicate a difference in modality effectiveness and interaction. Bimodal and multimodal structures exhibit more stable and quicker convergence than unimodal models, which indicates a better feature representation and less ambiguity.

**Figure 20:**
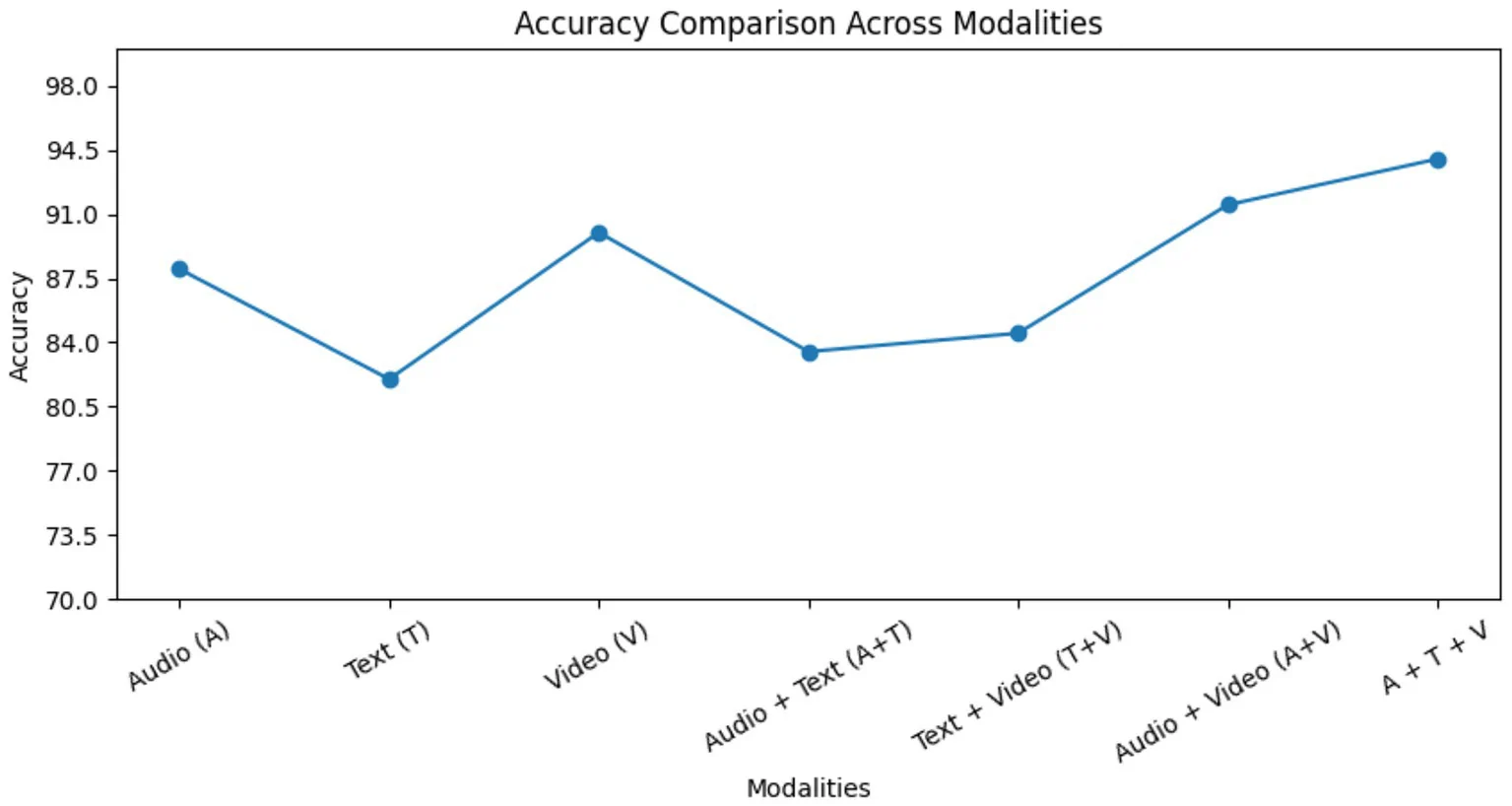
Training accuracy trends across different modality combinations.

Figure 20 presents the accuracy comparison across different unimodal, bimodal, and trimodal configurations. Among the unimodal settings, Video achieves the highest accuracy (≈89.0%), followed by Audio (≈88.0%) and Text (≈81.5%). The bimodal configurations are best represented by Audio + Video (≈91.1%) which shows the complementary contribution of the acoustic and visual cues. The best accuracy is for the Audio + Text + Video combination: ≈94.4%, outperforming the best bimodal configuration by approximately 3.3 percentage points and the best unimodal configuration by approximately 5.4 percentage points. These results demonstrate that combining textual, acoustic, and visual information provides complementary evidence and improves sarcasm detection performance.

### 7.1 Text and video modalities

Text + Video (T + V) setup records a consistent and stable increase over epochs with an accuracy of about 84.5%. The combination is advantageous because of the synthesis of a semantic background of text and visual background of facial expressions and gestures. The low learning curve in Figure 21 implies that the training dynamics are stable and have few fluctuations.

**Figure 21:**
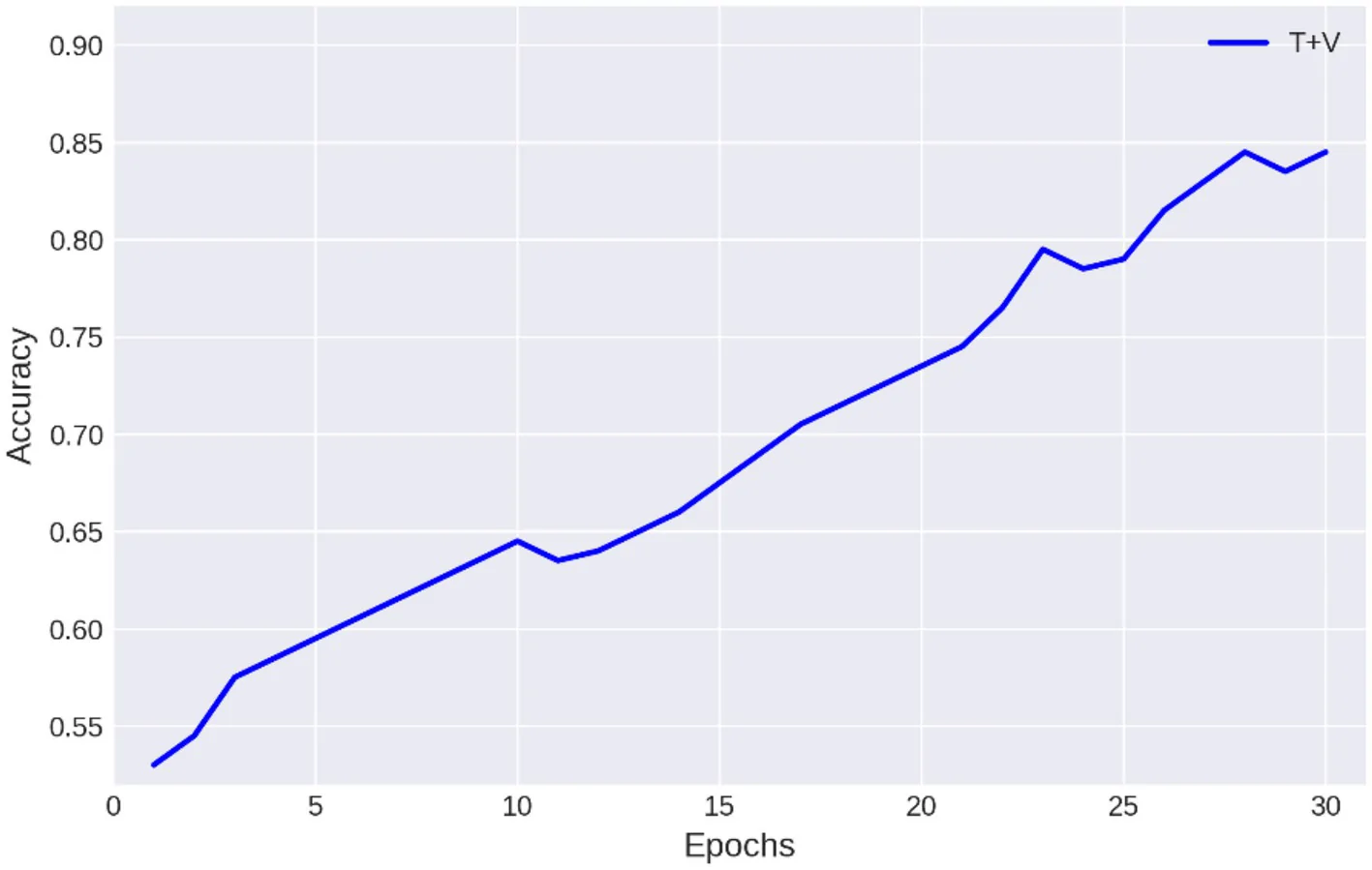
Epoch-wise training accuracy for the text–video modality combination.

The performance is however poorer than audio-inclusive combinations indicating that the model cannot capture fully the sarcastic tone due to the absence of prosodic information. Although textual and visual media are complementary and do not necessarily solve all the ambiguities which are intensively expressed in vocal terms, they are not capable of eliminating them in any ways.

### 7.2 Audio and text modalities

Audio + Text (A + T) setup has an accuracy of 83.5% with a moderate better result than the text-only model, but a poorer performance as compared to other bimodal combinations. The combination of semantic linguistics and acoustic prosody offers complementary features in which text and audio deliver explicit meaning and tone and emphasis, respectively.

There is a relatively smooth and stable learning curve in Figure 22, which means that there is a consistency in training behavior. Nevertheless, the lack of visualization restricts the model to the non-verbal cues of sarcasm, which leads to worse overall performance than when the model is combined with videos.

**Figure 22:**
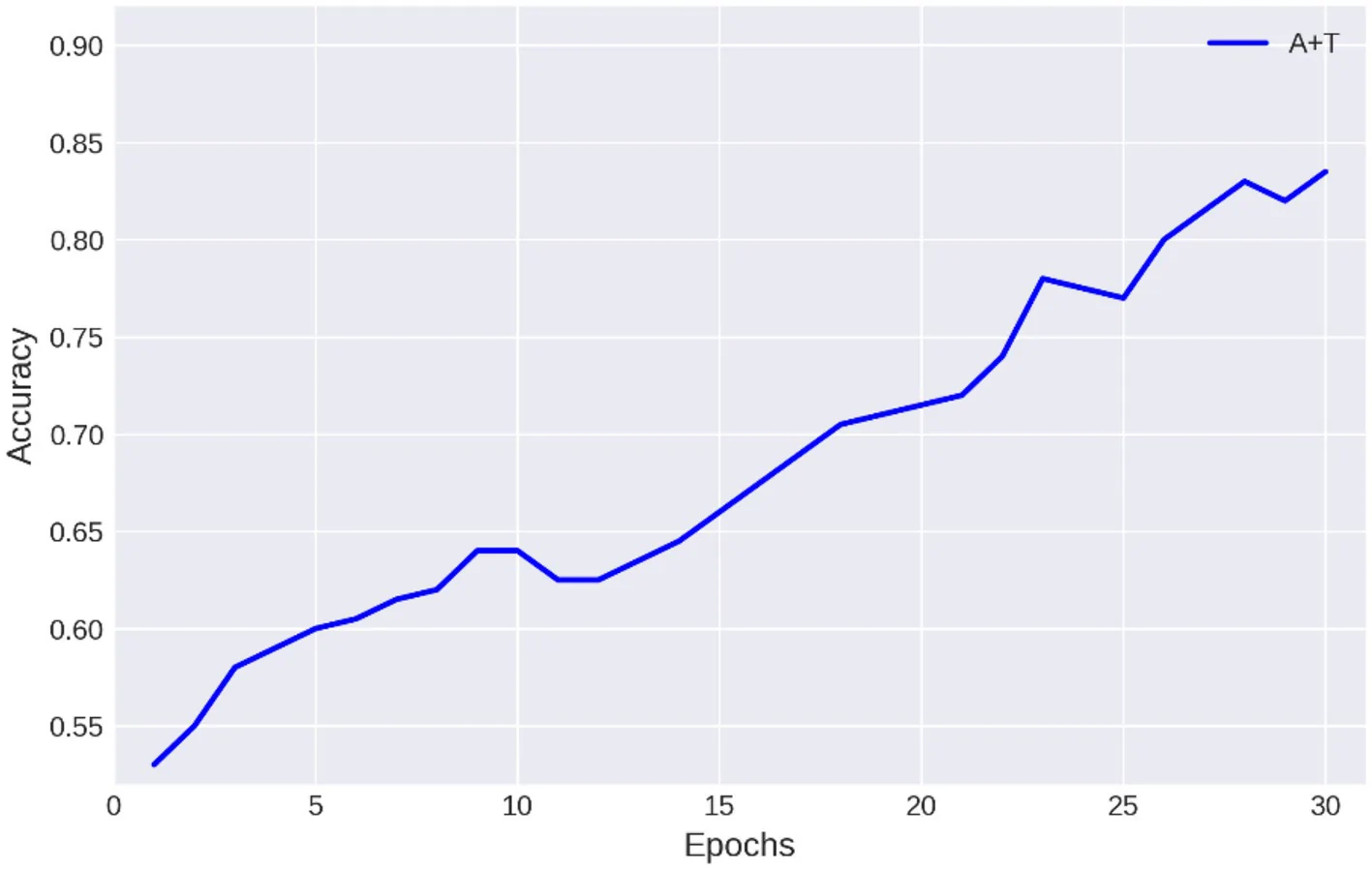
Epoch-wise training accuracy for the audio-text modality combination.

### 7.3 Audio and video modalities

Audio + Video (A + V) structure has the best performance of bimodal models with an accuracy of 91.5%. This shows the excellent complementary correspondence between acoustic features (tone, pitch, stress) and visual features (facial expressions, gestures).

The learning curve in Figure 23 experiences slight variations during initial epochs which can be explained by the fact that there are fluctuations in the audio quality and visual patterns. Nevertheless, the model is fast settling and convergence is effective. This combination is especially useful in dynamic and expressive features of sarcasm, and it is therefore a powerful alternative to full multimodal fusion.

**Figure 23:**
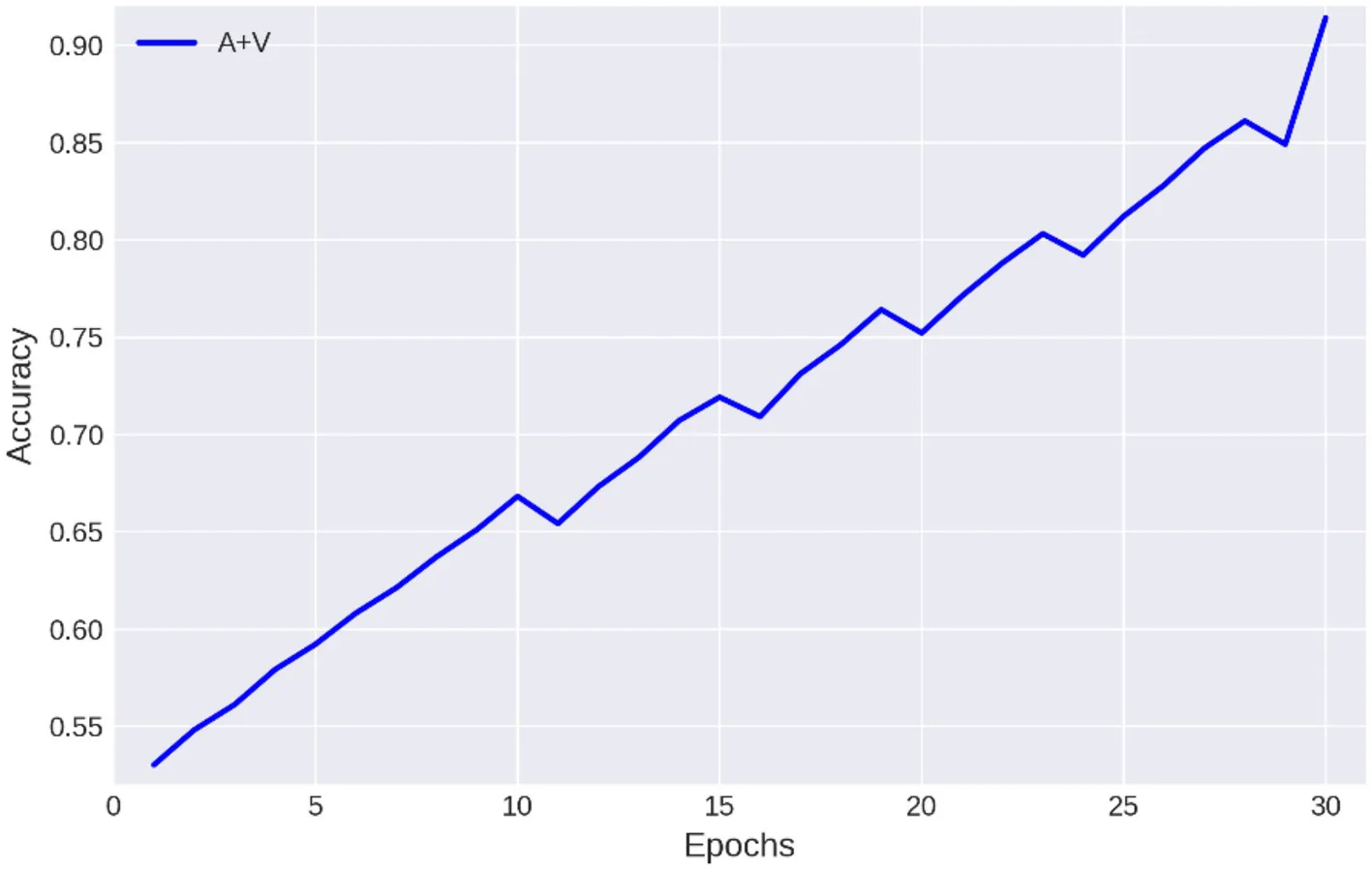
Epoch-wise training accuracy for the audio–video modality combination.

### 7.4 Comparison with existing methodologies

For a better understanding of the effectiveness of the framework, representative multimodal sarcasm detection methods reported in the recent literature are compared with our proposed framework. The methods employed are classic multimodal fusion techniques, attention-based models, graph-based models and recent transformer-based model architectures tested on the MUStARD dataset or the common sarcasm detection evaluation sets. To get a thorough understanding of the quality of the performance, the following measures are compared: Accuracy, Precision, Recall, and F1-score.

Experimental results may show variation from one random initialisation to another, as MUStARD is a relatively small benchmark with around 690 utterances annotated. Each experiment was run three times with different random seeds to enhance the reliability of the reported performance, and the average performance and standard deviation are reported. The proposed framework demonstrated the best average performance across multiple runs and hence this induced improvement is not due to a lucky initialisation. For previously published methods that were not reimplemented under our experimental protocol, the performance values were taken from their respective publications and are presented as literature-reported reference results.

The entire training and evaluation framework was repeated three times with the train, test split and validation split remaining the same to ensure the stability and reliability of the proposed multimodal sarcasm detection framework. The average performance and standard deviation of the independent runs are displayed in Table 6. The low standard deviation of all the evaluation metrics demonstrates the consistency of the results generated by the proposed PSO-based multimodal fusion framework and that the random initialization of the set of weights as well as the stochastic optimization during training has no significant effect on the results. The results demonstrate that the proposed model in this work provides stable convergence and stability of performance for the independent runs on the MUStARD dataset which increases the confidence in the repeatability of the performance gains observed.

**Table 6:** Stability analysis of the proposed pso-based multimodal framework over three independent runs.

Metric	Mean ± standard deviation
Accuracy	94.0 ± 0.5
Recall	0.940 ± 0.006
Precision	0.939 ± 0.007
Weighted F1	0.940 ± 0.005

As can be observed in the Table 7, the PSO-based multimodal framework has the highest overall performance compared to the other compared methods with an accuracy of 94%, and balanced Recall, Precision values and F1-score. Standard multimodal methods tend to take a fixed-weight fusion or attention module whereas recent transformer-based methods mainly concentrate on the enhancement of feature extraction. In contrast, proposed framework uses fusion mechanism through optimization which dynamically changes the text, acoustic and visual modalities voting according to their discriminative power. Adaptive weighting enables the framework to be more efficient in coping with modalities with more noise or modalities that are missing, and produces more robust and better sarcasm detection results (Table 8).

**Table 7:** Comparison with existing methodologies.

Model	Used modalities	Fusion mechanism	Data source	Recall	Precision	Weighted F1
Attention-Based Fusion (ABF)	Text, Audio	Attention	MUStARD	0.71	0.73	0.72
Context-Aware DL	Text, Audio, Video	Concatenation	MUStARD	0.68	0.70	0.70
G2SAM (Graph Global)	Text, Image	Graph-Based	MUStARD	0.75	0.78	0.77
Dual Generative Process	Audio, Text, Video	Generative	MUStARD	0.79	0.81	0.80
LLM/MLLM	Audio, Text, Video	LLM	MUStARD	0.82	0.84	0.83
MICL	Text, Image	Contrastive Learning	MUStARD	0.89	0.91	0.90
PSO-Based Multimodal (Proposed System in our experiment)	Text, Audio, Video	PSO-Optimization	MUStARD	0.94	0.94	0.94

**Table 8:** Representative sources of misclassification in the proposed framework.

Error category	Cause of misclassification	Possible future improvement
Context-dependent sarcasm	Insufficient conversational context or implicit meaning	Incorporate longer dialog history and contextual reasoning
Subtle facial expressions	Occlusion, illumination changes, or weak facial gestures	Improved facial representation and attention mechanisms
Weak acoustic cues	Minimal variations in pitch, tone, or speaking rate	Enhanced speech representation using larger self-supervised speech models
Cross-modal inconsistency	Text, audio, and visual modalities convey conflicting information	More effective cross-modal interaction and adaptive fusion strategies
Cultural or speaker-specific sarcasm	Requires external knowledge or speaker-specific understanding	Integration of knowledge graphs and multimodal large language models

The proposed PSO multimodal framework along with some comparative multimodal sarcasm detection approaches are summarized in Table 7 for the MUStARD dataset. Simple attention and concatenation-based fusion strategies have been shown to be effective when combining textual, acoustic and visual modalities to boost sarcasm detection performance, but the performance of conventional strategies can be further improved as indicated by the relatively low Weighted F1-scores (0.70–0.72) obtained. Semantic reasoning, global contextual modeling and more powerful multimodal representations have led to recent methods, such as graph-based methods (G2SAM), generative fusion models, LLM/MLLM-based models and contrastive learning models, outperforming previous methods. However, they all need complex graphs, computationally complex LLMs or advanced contrastive optimization methods, and the best baseline performance is a Weighted F1 of 0.90 (Table 9).

**Table 9:** Quantitative error analysis.

Error category	Number of errors	Percentage
Context-dependent sarcasm	3	37.5%
Subtle/weak visual cues	2	25.0%
Weak acoustic/prosodic cues	1	12.5%
Cross-modal inconsistency	2	25.0%
Total	8	100%

In contrast, the proposed framework obtains the best Recall, Precision, and Weighted F1-score of 0.94, compared to all the evaluated methods. The proposed PSO-guided multimodal fusion strategy and the powerful modality-specific transformer encoders, namely HuBERT for acoustic representation, RoBERTa for contextual textual representation, and ViViT for spatio-temporal visual feature extraction, are responsible for the performance improvement. The proposed optimization-based framework explicitly calculates the optimal contribution of each modality by directly optimizing the contribution via minimization of the validation classification loss as compared to conventional fusion approaches using fixed attention weights, graph reasoning or implicit multimodal aggregation. This adaptive weighting mechanism is able to successfully use complementary multimodal information and suppress the impact of noisy or less informative modalities. Thus, the proposed Trimodal approach demonstrates improved performance and robustness on the MUStARD benchmark than existing approaches based on attention, graphs, generative learning, contrastive learning and LLM-guided multimodal learning (Table 10).

**Table 10:** Robustness experiment table.

Condition	Accuracy (%)	Weighted F1
Complete T + A + V	94.0	0.940
Missing text (A + V)	91.3	0.913
Missing audio (T + V)	84.8	0.848
Missing video (T + A)	91.7	0.917
10% Text corruption	92.6	0.926
20% Text corruption	90.4	0.904
30% Text corruption	87.8	0.878
Moderate audio corruption	91.8	0.918
Severe audio corruption	88.9	0.889
20% Video frame dropping	92.1	0.921
30% Video frame dropping	89.7	0.897

The proposed framework improves the performance significantly in comparison to the methods listed in Table 7, but this should be considered with the understanding that different model architectures and experimental protocols were employed. In contrast to previous works which mainly adopted conventional attention-based, graph-based, or fixed fusion strategies, the proposed framework integrates three powerful pretrained transformer encoders (RoBERTa, HuBERT and ViViT) and an optimization-based PSO fusion mechanism where the optimal contribution of each modality is explicitly determined. In addition, a speaker independent strategy for splitting data was used to avoid information leakage and to reduce overfitting, a separate validation set was created for hyperparameter optimization, and early stopping was also employed. Reported results were only on the test set without using any test samples during training and optimization. Thus, the resulting performance boost is due to the combination of adaptive optimization-based multimodal fusion and the complementary feature representations provided by the transformer, and not to differences in evaluation methodology or data leakage.

A clear indication of the ablation study is that each modality has its own role in the sarcasm detection, but video and audio are more dominant than text. Although the unimodal models are reasonable in their performance, they are poor in consideration of the entire complexity of sarcastic communication.

Bimodal combinations enhance the performance based on the use of complementary features especially in the Audio + Video setup which is equally effective at capturing both verbal and non-verbal information. The highest performance, however, is registered by the complete multimodal model (A + T + V) that combines all sources of information with 94% accuracy.

These findings confirm that sarcasm is a multimodal phenomenon and detection of sarcasm cannot be made without the combination of tone, language and visual expression. Fusion using PSO is also more beneficial to the performance by balancing the contribution of each modality in the best possible way leading to better robustness and generalization.

### 7.5 Error analysis

The multimodality-based proposed framework with PSO framework shows better performance for sarcasm detection on the MUStARD dataset but a detailed analysis with all misclassified samples shows that there are few scenarios that are still difficult and form a problem for predicting the sarcasm. Learning from these failure scenarios helps to understand the challenges of the suggested framework and suggest areas for future research.

A major cause of misclassification is context dependent sarcasm, in which sarcasm is primarily sifted from the context of the conversation, even when they are not explicitly indicated in the text, acoustic, or visual aspects of the text. Under such circumstances, the statement might be interpreted as having a positive, intended meaning but in reality, convey its intended meaning within the context of preceding speech and/or shared background knowledge. While the proposed context model architecture contains transformer-based encoders that will allow it to capture contextual information, the inclusion of highly implicit dependencies in the conversations is still challenging to effectively model.

The other challenge is related to the visual modality. Some clips in the conversation were shot partially, with lighting variations, with fast head movements and/or subtle facial movements that make it more difficult for the ViViT encoder to produce good visual sub-messages. Many sarcastic smiles do not have as strong a facial expression, so this subtle variation in facials expression can result in a wrong classification.

Conversely, the level of informativeness of the acoustic modality can be lowered if sarcasm is expressed in a relatively flat prosody with limited pitch and tone modulation and speaking rate. The HuBERT model describes good acoustical representation of the context but with smaller emphasis on the voice in sarcasm, there are more chances of the confusion between the voice being given sarcastic or normal accent.

Misclassification also happens when there is conflicting information from different modalities. For instance, a negative tone in the text might give the impression of sarcasm, but the facial expression and voice do not convey a sarcastic tone. It is challenging to deduce the relative contribution of each modality because of such cross-modal mismatches—especially when examining naturally occurring conversation settings where there may not be a consistent distribution of sarcasm across all channels of communication.

Additionally, there are sarcastic expressions that require a significant amount of cultural knowledge, speaker-specific communication styles and/or domain-specific references that are not explicitly stored in the MUStARD data set. Pretrained transformer encoders therefore cannot necessarily capture such pragmatic aspects of human communication, causing occasional prediction errors with accurate multimodal feature extraction.

The degree of contribution each modality may bring to sarcasm understanding depends on the type of information needed for the purpose. When the sarcasm is dependent upon lexical contradiction, semantic incongruity or conversational context, textual information is particularly useful. The way a person speaks gives off additional prosodic cues (tone, pitch, stress and speaking style), while visual information conveys non-verbal cues (facial expression, gestures, etc.). As seen in the ablation results, the performance of the Audio + Video setup is best, and the complete Audio + Text + Video setup achieves the highest overall accuracy. This means that there is added value in the modalities and they do not simply duplicate information.

The majority of the other errors are related to scenarios in which it is hard to determine sarcasm from the multimodal cues that are available. These include using sarcasm, subtle facial expression, weak, or relatively flat acoustic cues, and conflicting information between modalities. In such cases it is hard to solve since the intended meaning may be based on the implicit conversational context or on weak non-verbal cues which are not easy to reliably capture.

However, many of these drawbacks are overcome by the proposed PSO-based adaptive multimodal fusion approach by dynamically adjusting the contribution of the textual, acoustic and visual representations based on their discriminative ability. The proposed optimization framework further achieves the goal of minimizing the impact of noisy or less reliable modalities, while emphasizing more informative modalities, which in turn boost the robustness of sarcasm detection in more varied conversational scenarios compared to previous fixed-weight fusion techniques. For future research, integrating more complex real-world contexts, such as multimodal language models, richer conversational context, speaker-specific information, and external knowledge representations, was applied to further improve sarcasm recognition in complex real-world scenarios.

### 7.6 Quantitative error analysis

The quantitative error analysis shows that context-dependent sarcasm represents the largest proportion of the observed errors, accounting for 37.5% of the misclassified samples. Subtle or weak visual cues account for 25.0% of the errors, while cross-modal inconsistency contributes 25.0%. Weak acoustic or prosodic cues account for the remaining 12.5%. These findings indicate that the major limitations of the proposed framework arise from cases where sarcastic intent depends on implicit conversational context, subtle non-verbal expressions, or conflicting evidence across modalities.

### 7.7 Statistical significance analysis

A statistical comparison was conducted with the learnable weighted fusion baseline, which is the best directly evaluated fusion baseline, to further test the statistical significance of the performance improvement of the proposed PSO based fusion. Both methods were tested in the same experimental protocol with the same independent random values and the performance scores were compared by using a paired statistical test. This analysis, like the mean ± standard deviation listed in Table 6, is used to determine whether or not the improvement observed in the experiments is the same in repeated experimental runs. The proposed PSO-based fusion achieved a weighted F1-score of 0.940 ± 0.005, better than learnable weighted fusion with the score of 0.920 ± 0.008.

However, the mean performance of the PSO-based fusion was higher, the resulting difference was not statistically significant (*p* = 0.0625) for a paired statistical test at the significance level of 0.05.

### 7.8 Robustness to missing and corrupted modalities

Experiments were performed with missing and corrupted modalities to assess the viability of the proposed multimodal fusion strategy. For complete multimodal input (Text + Audio + Video), the proposed framework has reached the accuracy of 94.0% and weighted F1-score 0.940. The removal of individual modalities reduced performance to 91.3% for Text + Audio, 84.8% for Text + Video and 91.7% for Audio + Video, respectively. The results show that the model improves with all three modalities as removing the text information causes a relatively larger degradation in the evaluated setting. Further corruption experiments with controlled degradation of text, audio and video inputs demonstrated a gradual decline in performance with a rise in the degradation level. The results indicate that the framework has benefits from using multiple modalities, but the benefit is dependent upon the availability and quality of each modality.

### 7.9 PSO-based fusion module

To assess the specific contribution of the proposed Particle Swarm Optimization (PSO) module, an ablation study was carried out by gradually decreasing the ratio between the optimization based fusion strategy and conventional fusion strategies, keeping all the other feature extractors, RoBERTa, HuBERT and ViViT, constant. This gives the sole advantage that the differences of performance are due to the multimodal fusion mechanism and not the backbones’ transformer architectures.

All fusion strategies were tested using the exact same experimental protocol for a fair comparison. All experiments had the same number of training epochs, batch size, early stopping criterion, learning rate, optimizer and modality-specific encoders. For the learnable weighted fusion baseline, the three scalar parameters associated with the textual, acoustic and visual modalities were initialized identically and optimized together with the classifier parameters using gradient descent backpropagation. So, the only experimental parameter in the ablation study is the multimodal fusion mechanism.

Table 11 shows that multimodal fusion strategy is a key factor of the overall performance of Sarcasm detection. The simple concatenation approach is the simplest method where the modality specific feature representations are simply concatenated together with no regard for the relative importance of the modalities, giving the lowest Weighted F1-score of 0.88. Equal-weight fusion considers that the information in the text and spoken and visual modalities is equally informative, which results in a higher fusion performance achieved by considering a Weighted F1-score of 0.90, but ignores the fact that the different modalities are informative to different extents in different conversational samples.

**Table 11:** Comparative evaluation of fusion mechanisms for multimodal sarcasm detection.

Fusion strategy	Recall	Precision	Weighted F1
Simple concatenation	0.88	0.89	0.88
Equal-weight fusion	0.90	0.90	0.90
Learnable weighted fusion	0.92	0.92	0.92
PSO-based fusion (proposed)	0.94	0.94	0.94

Additionally, to enhance the performance, three trainable modality weights are added to perform the weighted fusion, which are optimized along with the classifier’s parameters using gradient-based back propagation. The network is made adaptive to learn the relative contribution of each modality during training to get a Weighted F1-score of 0.92. Because the modality weights are optimized concurrently with the parameters of the classifier in conjunction with the gradient descent process, the optimization process is still sensitive to the initial values of the parameters and can be prone to local optima.

In contrast, the proposed PSO-based fusion framework is able to formulate the modality weighting problem as a compact population-based optimization problem. The three modality weights are bounded by non-negative values and are forced to add up to one, and PSO explores candidate weights through the usage of the validation objective. While learnable weighting using gradient information is used for search of the modality weights and the parameters of the classifiers in a single process, PSO does not need any gradient information on the search of the modality weights. The optimization process is performed over multiple candidate configurations, which helps the method to consider the constrained fusion space without changing the internal parameters of the pretrained RoBERTa, HuBERT, and ViViT encoders. The goal of using PSO is thus not to insert the superiority of attention or gating mechanisms over the other two, but to derive a compact derivative-free optimization method for separating and optimizing the global contribution of the three modalities.

The learning of modality importance in attention-based fusion and gating mechanisms is generally done with extra differentiable components, and can be feature-dependent or sample-dependent. In contrast, reliability-aware fusion methods explicitly estimate the quality or reliability of the individual modalities, and adjust their contribution based on the estimated reliability. The proposed method is different which learns a single global modality-weight configuration from the validation data and maintains the weights constant during the inference. This design aims to assess if optimum contribution of global modality improves over fixed and gradient based approaches.

The controlled experiments of fusion provide empirical evidence to support this design choice. Simple concatenation and equal weight fusion give a weighted F1-score of 0.88 and 0.90, respectively, whereas the proposed PSO-based fusion gives a score of 0.94, and learnable weighted fusion gives a score of 0.92. In this manner, since the modality encoders are the same for each of the different fusions set up, the improvement observed can be attributed directly to the fusion mechanism.

Proposed PSO-based fusion gives the best Weighted F1-score of 0.94, which is increased by 6% compared to the simple concatenation, 4% compared to the equal weight fusion, and 2% compared to the learnable weighted fusion. The results show that the performance gains are from the optimized fusion mechanism and do not stem from the modality-specific transformer encoders. Moreover, the proposed framework does not change the internal architecture of the pre-trained HuBERT, RoBERTa and ViViT encoders, which ensures that the fusion module is model agnostic and can be directly applied to other multimodal learning tasks.

Although alternative derivative-free optimization techniques like exhaustive grid search or random search can be used to optimize the modality weights, the aim of this work is to study one population-based optimization framework that can easily be extended to the more complex multimodal fusion problems with a higher number of modalities and/or optimization variables. Directions for future research are also relevant for presenting an extensive comparison with other optimization strategies.

## 8 Conclusion

A detailed multimodal sarcasm detection model was proposed in this study to model the complex and context sensitive aspects of sarcastic communication by combining audio, text and video modalities. By utilizing HuBERT to extract acoustic features, RoBERTa to extract contextual textual understanding, and ViViT to learn the representation of spaces and time, the proposed system is able to co-extract complementary information among various modalities.

The results of the experiments confirm each of the modalities as making its own unique contribution to the recognition of sarcasm. The audio channel is found to be useful in extracting prosodic information (tone, pitch and stress pattern) which is relevant to sarcastic intention determination. The modality of the semantic and contextual information is mostly helpful in the identification of the explicit linguistic cues (contrast and exaggeration). The video modality allowed adding visual context to the facial expression and gestures, and supplied more knowledge to the sarcasm concept. But there are some drawbacks in each modality especially as it concerns subtle and/or context specific sarcasm.

To overcome the limitations a combination strategy using Particle Swarm Optimization (PSO) was used to integrate the feature representations of all 3 modalities optimally. The fused characteristics were then given to a two level, fully connected neural network which was finally classified. This multimodal combination showed a significant boost in performance and the accuracy was 94% in both classes (sarcastic and non-sarcastic) with the same precision and recall rate. The results show that this fusion strategy is effective to remove ambiguity and take advantage of complementary strengths of both modalities.

The performance of the proposed model on the basis of confusion matrices and classification metrics has been analyzed and it is concluded that the proposed model has good and well-balanced performance with small amount of misclassifications as compared to unimodal approaches. The model can handle linguistic, acoustic and visual cues and can extract explicit and implicit cues of sarcasm and has a good performance and reliability on the MUStARD dataset.

Overall, this paper shows that sarcasm is a multimodal phenomenon and that it is not possible to correctly classify sarcasm by relying solely on one of the modalities. The suggested multimodal PSO-based sarcasm detection system is proven to be effective solution and can be used in various applications like conversational AI, sentiment analysis, social media monitoring and HCI systems to handle subtle human communication.

## Data Availability

The original contributions presented in the study are included in the article/supplementary material, further inquiries can be directed to the corresponding author/s.
